# Semantic matching in GUI test reuse

**DOI:** 10.1007/s10664-023-10406-8

**Published:** 2024-05-09

**Authors:** Farideh Khalili, Leonardo Mariani, Ali Mohebbi, Mauro Pezzè, Valerio Terragni

**Affiliations:** 1 Northeastern University, Boston, MA USA; 2 University of Milano - Bicocca, Milan, Italy; 3 USI Universitá della Svizzera italiana, Lugano, Switzerland; 4 Constructor Institute Schaffhausen, Schaffhausen, Switzerland; 5 University of Auckland, Auckland, New Zealand

**Keywords:** Software testing, Testing Android apps, Test reuse, Semantic matching for testing, Automatic test generation, Word Mover, Universal sentence encoder, Word2vec, Glove, Fast

## Abstract

Reusing test cases across apps that share similar functionalities reduces both the effort required to produce useful test cases and the time to offer reliable apps to the market. The main approaches to reuse test cases across apps combine different semantic matching and test generation algorithms to migrate test cases across Android apps. In this paper we define a general framework to evaluate the impact and effectiveness of different choices of semantic matching with Test Reuse approaches on migrating test cases across Android apps. We offer a thorough comparative evaluation of the many possible choices for the components of test migration processes. We propose an approach that combines the most effective choices for each component of the test migration process to obtain an effective approach. We report the results of an experimental evaluation on 8,099 GUI events from 337 test configurations. The results attest the prominent impact of semantic matching on test reuse. They indicate that sentence level perform better than word level embedding techniques. They surprisingly suggest a negligible impact of the corpus of documents used for building the word embedding model for the Semantic Matching Algorithm. They provide evidence that semantic matching of events of selected types perform better than semantic matching of events of all types. They show that the effectiveness of overall Test Reuse approach depends on the characteristics of the test suites and apps. The replication package that we make publicly available online (https://star.inf.usi.ch/#/software-data/11) allows researchers and practitioners to refine the results with additional experiments and evaluate other choices for test reuse components.

## Introduction

GUI applications offer rich sets of GUI interactions that often result in huge execution spaces. A main challenge of testing GUI applications consists of effectively sampling the execution space, to thoroughly test the application within time and budget constraints. Automatic test case generators (Google 2017; Machiry et al. 2013; Amalfitano et al. 2012; Anand et al. 2012; Ermuth and Pradel 2016; Mirzaei et al. 2015; Gu et al. 2019; Mao et al. 2016; Dong et al. 2020) largely reduce the human effort required to generate GUI test cases; However they generate many test cases that execute meaningless combinations of actions thus missing combinations of events that can reveal faults, and rely on implicit oracles (Memon et al. 2003a; Moran et al. 2016; Zhao et al. 2019) that cannot reveal failures related to the semantics of the app under test.

The recent Test Reuse approaches that automatically migrate GUI test cases across similar applications (Behrang and Orso 2019; Lin et al. 2019; Rau et al. 2018b, a; Behrang and Orso 2020; Qin et al. 2019) address the main limitations of automatic test case generators, by inheriting the purpose and effectiveness of test cases across applications.

They generate a test case for a new application, hereafter *target application*, from a test case of an already tested applications, hereafter *source application*. Test Reuse approaches generate semantically relevant *target test cases*, that is test cases for *target applications* to be tested, by adapting *source test cases*, that is test cases designed to effectively test *source applications*.

Test Reuse approaches can effectively migrate test cases across applications that share some functionalities, which is a common practice in many domains, and everyday routine in many cases, like in the the extremely relevant domain of mobile apps: Hu et al. report that 196 (63.4%) of the top 309 non-game mobile apps in the Google Play Store can be clustered into 15 groups, each sharing many common functionalities (Hu et al. 2018); Ebrahimi et al. classify the 1.8M apps of Apple App Store in 23 categories, and the 2.87M apps of Google Play in 35 distinct categories (Ebrahimi et al. 2021). In this paper, we target Test Reuse approaches for the extremely relevant domain of Android applications.

The currently available Test Reuse approaches for Android apps are ATM (Behrang and Orso 2019), CraftDroid (Lin et al. 2019) and AdaptDroid (Mariani et al. 2021). These three approaches successfully migrate non-trivial test cases, showcasing the potential of Test Reuse.

They combine a Semantic Matcher procedure with a Test Generator. The Semantic Matcher procedures identify pairs of semantically similar events between the source and target apps, by recognizing semantic similarities among the textual descriptors associated with the GUI widgets by means of word embedding techniques (Mikolov et al. 2013b). The Test Generator exploits the similarities between events to generate the target test cases, according to specific strategies: ATM and CraftDroid sequentially migrate the events in the source test case, while AdaptDroid uses a search-based process to derive the migrated test.

The usefulness of Test Reuse approaches strongly depends on the effectiveness of semantic matching: Pairing semantically similar events is a necessary condition for successful migration, regardless of the implemented migration strategy. Thus, assessing the impact and effectiveness of semantic matching both in isolation and in the Test Reuse process is extremely important to improve Test Reuse approaches. Zhao et als FrUITeR framework (Zhao et al. 2020) empirically compares Test Reuse approaches as a whole; However there exist no frameworks to assess the effectiveness of semantic matching techniques in the context of Test Reuse, and their impact on the Test Reuse process.

In this paper, we present the first complete empirical study on both the effectiveness of semantic matching of GUI events in the context of Test Reuse and the impact of semantic matching on Test Reuse. We identify four main components that characterize the semantic matching procedures of all Test Reuse approaches for Android apps: Corpus of Documents, Word Embedding, Event Descriptor Extractor, Semantic Matching Algorithm, and we comparatively evaluate the impact of the different choices for each component. The study offers a thorough empirical comparison of the impact of the various implementations of the semantic matching components on Test Reuse approaches, and proposes SemFinder, a new Semantic Matching Algorithm that outperforms existing ones.

The results reported in this paper show that SemFinder finds similar events that other approaches miss, thanks to an original combination of the way SemFinder both selects events for matching and uses semantic matching. Thus, SemFinder reuses a larger set of test cases than the state-of-the-art approaches. For instance, the running example that we introduce in Section 2 shows a test case that only SemFinder correctly migrates from a source app *Money Tracker* to a target app *EasyBudget*. All state-of-the-art approaches do not generate valid test cases, since they do not select the suitable candidate events, as discussed in this paper.

In this paper, we study semantic matching of GUI events both in isolation and in the context of Test Reuse, that is, independently from the Test Reuse process, and with respect to the quality of the migrated test cases, respectively. We report the results of a study of semantic matching in isolation on 8,099 GUI events by 337 semantic matching configurations from 30 Android applications, thus offering important evidence for defining effective semantic matching procedures. We report the results of a study of semantic matching in the context of both the ATM and CraftDroid Test Generator on 6,000 test migrations from 68 configurations. The results that we report in this paper show that (i) Semantic Matching Algorithm and Event Descriptor Extractor are the most impactful components on Test Reuse; (ii) sentence level word embedding techniques (Word Mover, Universal Sentence Encoder) perform better than word level ones (Word2vec, Glove, Fast); (iii) semantic matching of events of a carefully selected subset of types performs better than semantic matching of events of all types.

The work presented in this paper extends our preliminary study (Mariani et al. 2021) by (i) extending the study of semantic matching in isolation with the Semantic Matching Algorithm of AdaptDroid, that was not included in the former study, (ii) presenting the empirical results of a large study of semantic matching in the context of Test Reuse (research questions *RQ3-RQ6*) on 68 semantic matching configurations of 29 mobile applications, 89 test scenarios, and more than 6,000 migrated test cases (iii) defining a publicly available framework to assess semantic matching and its components, both in isolation and in the context of Test Reuse^1^, (iv) discussing new relevant findings that extend the empirical evidence about the impact of semantic matching in Test Reuse, and (v) identifying a detailed and rigorous presentation of both the semantic matching components and their role in the Test Reuse process.

This paper contributes to the research on Test Reuse approaches by

A) identifying the core components and the workflow that characterize all Test Reuse approaches,
B) defining a publicly available framework to evaluate the semantic matching of GUI events both in isolation and in the context of Test Reuse,
C) defining and evaluating SemFinder, a new Semantic Matching Algorithm that outperforms the state-of-the-art approaches
D) presenting and discussing the results of the empirical evaluation of semantic matching in isolation on 337 configurations,
E) presenting and discussing the empirical evaluation of semantic matching in the context of Test Reuse with both the ATM and CraftDroid Test Generator on 68 semantic matching configurations,
F) discussing several findings that influence future research and practice in Test Reuse.

The results presented in this paper help both testers optimize the testing effort and researcher refocus the research plans. The evaluation framework, Test Migration Evaluator, and the Semantic Matching Algorithm, SemFinder, that we present in this paper, indicate the strengths and limitations of test migration approaches, and largely improve automatic test migration, respectively. They lay the foundation for effectively and efficiently migrating test cases. Testers can optimize the testing effort by automatically migrating test cases from similar apps, and focusing on the new features of the apps. The results of the empirical evaluation presented in this paper reveal a substantial gap that remains between the best semantic matching configuration and the perfect matching, despite the relevant progresses with respect to the pioneer work. It shows the researchers the main research directions to further improve test reuse.

This paper is organized as follows. Section 2 introduces the terminology, and illustrates the Test Reuse approach. Section 3 presents the core components that characterize the workflow of the different Test Reuse approaches proposed in the literature, presents the main choices for each component, discusses the choices that characterize existing approaches to Test Reuse, and introduces SemFinder, an Semantic Matching Algorithm that we define in this paper. Section 4 presents the results of an extensive experimental campaign that we carried on to answer six research questions. Section 5 discusses the main related work. Section 6 summarizes the main contribution of this paper, and highlights the research directions that the results reported in this paper open.

## Test Reuse Approach

Our study focuses on Test Reuse of Graphical User Interface **(GUI)** applications. In particular, we target GUI applications for the Android platform as current GUI Test Reuse approaches target Android apps. However, the idea of GUI Test Reuse could be applied to any pairs of GUI apps that share similar functionalities. Indeed, most of the concepts and definitions presented in this paper are general enough to be applied to GUI applications belonging to other platforms.

A GUI is a forest of hierarchical windows where only a window is active at any time (Memon et al. 2003b). Windows host **widgets**, which are atomic GUI elements characterized by attributes (such as, *text* and *resource-id*). At any time, the active window has a **state** $\textbf{S}$ that encompasses the attribute values of the displayed widgets. Some widgets expose user-actionable events to let users interact with the app (Dix 2009). An **event** is an atomic interaction on a widget. *GUI events* are human-computer interactions, for instance, click on widgets of type *Button*, or fill widgets of type *EditText*. Following previous Test Reuse approaches, we abstract the implemented widget type and group GUI events into two types: *clickable* and *fillable*. *Oracle events* are checks on the state of the widgets, for instance *exist(text)*. More formally, an event is a triple $\langle widget, type, input\rangle$. Widget indicates the GUI element the event is executed on. Type can be clickable, fillable or oracle. Input is the string that fills a widget, if it is a fillable event, the string that needs to be checked, if it is a an oracle event, empty otherwise.

A **GUI test t** is an ordered sequence of events $\langle e_1,..., e_n\rangle$ on widgets of the active windows. A test execution induces a sequence of state transitions $S_{0} \xrightarrow {e_1} S_1 \xrightarrow {e_2} S_2 \ldots \xrightarrow {e_n} S_{n}$, where $S_{i-1}$ and $S_i$ denote the states of the active window before and after the execution of $e_i$, respectively. A GUI test case can have one or more assertion oracles that check the correctness of the state $S_i$ obtained after the execution of an event $e_i$ (Barr et al. 2015). For example, by checking for the absence or presence of widgets with specific attributes values.

Test Reuse approaches for GUI applications (Zhao et al. 2020) automatically migrate GUI test cases (including oracles) across apps that share similar functionalities. More formally, given two apps $A^{s}$ (source) and $A^{t}$ (target), and a “source” test $t^{s}$ for $A^{s}$, Test Reuse approaches generate “target” test case $t^{t}$ that tests $A^t$ as $t^s$ tests $A^{s}$. They create $t^{t}$ by searching $A^t$ for events that are semantically similar to events in $t^{s}$.

**Fig. 1:**
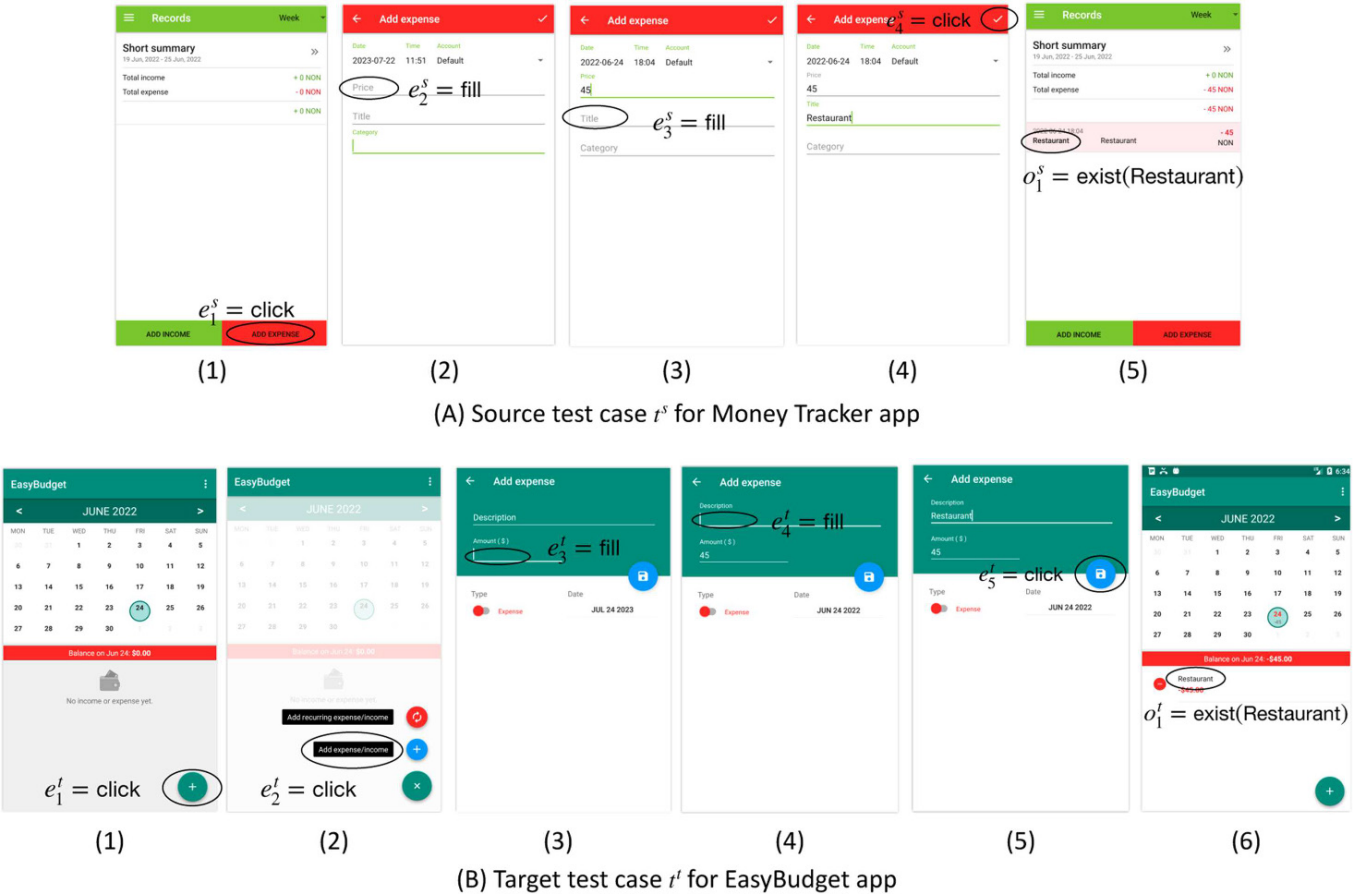
Test reuse example, the target test case (B) is obtained by migrating the source test case (A)

Figure 1 shows an example that we have taken from the experiments of ATM, and that we use as running example through the paper. The figure shows a migration from a test designed for the source app *Money Tracker* (A) to the target app *EasyBudget* (B). The two test cases verify the same feature, namely adding an expense item. The migration process generates the test case for the target app *EasyBudget* by finding the events that correspond to the events in the test case of the source app *Money tracker*. It determines the events of the target app that correspond to the events in the source app through a semantic similarity relation $\sim$. It completes the sequence of corresponding events with additional events that are needed to produce a feasible test, and that we refer to as ancillary events later in the paper. In the example the migration process finds two pairs of clicks with semantically similar attributes ($e^s_1 \sim e^t_2$ and $e^s_4 \sim e^t_5$), two pairs of events that fill fields with similar semantics ($e^s_2 \sim e^t_3$ and $e^s_3 \sim e^t_4$), and a pair of events with similar text attribute ($e^s_4 \sim e^t_5$). It generates the test case for the target app *EeasyBudget* by completing the sequence of events that correspond to the events in the source app *Money Tracker* with an ancillary event ($e^t_1$) that is needed to build a feasible test case for *EeasyBudget*.

Current Test Reuse approaches define the semantic similarity relation $\sim$ as a one-to-one mapping between a source and a target event. The notion of semantic similarity of GUI events largely influences the ability of Test Reuse techniques to recognize corresponding events, thus impacting on the whole migration process.

**Fig. 2:**
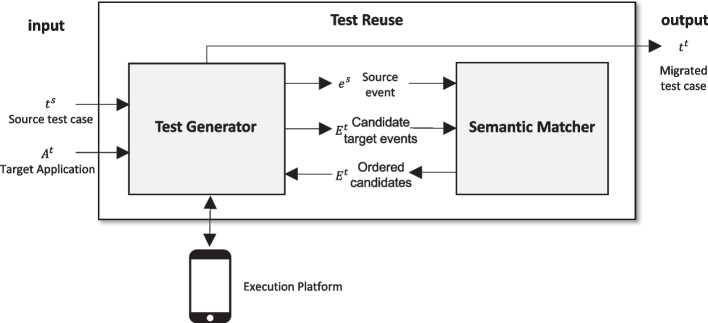
Test Reuse Overview

Figure 2 overviews the GUI Test Reuse process, which is shared by all current techniques. GUI Test Reuse combines semantic matching of GUI events with test generation. Semantic matching of GUI events identifies semantically similar events across source and target apps. Test generation exploits the similarities identified with semantic matching to migrate GUI test cases from the source to the target app. More specifically, Test Reuse can be abstracted into two coarse grain components: Test Generator and Semantic Matcher. We give a detailed description of the two components in Section 3.

Test Reuse approaches need to match semantically similar GUI events across apps. Such a semantic matching should capture the event semantics, while abstracting the implementation details. Indeed, two different apps might implement the same logical action with different widgets (for instance, a button in one case and an image button in another). Intuitively, Test Reuse approaches aim to generate test cases for the target app that maximize the number of *semantically similar events*, possibly in the order prescribed by the source test.

Current approaches characterize the semantics of events by relying on the textual attributes found in the GUI. In particular, they associate each event with its *descriptor* that encompasses the textual attributes of the widget associated with the event. For instance, both events $e^s_3$ and $e^t_4$ in *Money Tracker* and *EasyBudget* example in Fig. 1 are associated with an attribute *neighbor-text* with values *“Title”* and *“Description”*, respectively. They then identify similar semantics by querying a Word Embedding Model that recognizes words or sentences that express similar concepts. For instance, a Word Embedding Model would recognize that *“Title”* and *“Description”* are semantically similar.

## Test Reuse Workflow

In this section, we define a Test Reuse workflow that characterizes Test Reuse across Android applications. We discuss the implementation of the different components, and report the choices that we used in our experimental study. We indicate the alternative implementation of each component, and discuss the choices that characterize the current approaches, ATM, CraftDroid and AdaptDroid. The general workflow allows us to identify and compare combinations of different choices for each component, and identify SemFinder, a new Semantic Matching Algorithm that supersedes current approaches.

**Fig. 3:**
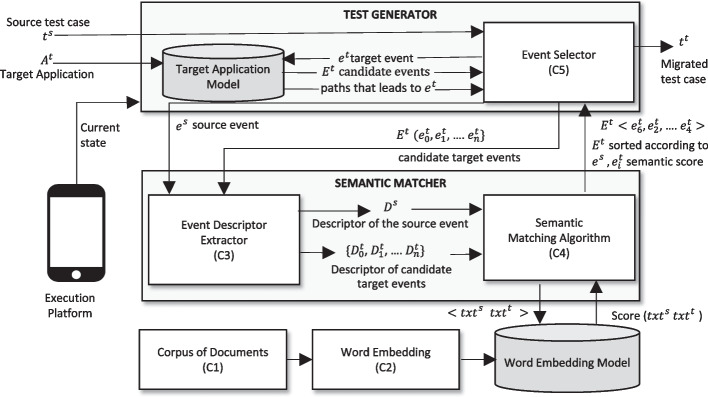
Test Reuse Workflow

Figure 3 shows the workflow of Test Reuse. Given a test case $$t^s = \{ e^s_0, e^s_1, \cdots e^s_n\}$$ from a source application, the Test Generator explores the target application $$A^t$$ to find a match for each event in $$t^s$$, and it generates a test case $$t^t$$ for the target application. The Test Generator retrieves a set $$E^t~=~\{ e^t_0, e^t_1, \cdots e^t_n\}$$ of candidate events from both the current state and Target Application Model, and queries the Semantic Matcher to sort events according to their similarity with the events $$e^s \in t^s$$. The Semantic Matcher sorts the candidate events $$ e^t_i$$ according to the similarity score $$\langle e^s, e^t_i \rangle $$ that it computes by aggregating the scores that it retrieves from the Word Embedding Model for each pair of attributes in the events descriptors ($$Score(txt^s,txt^t)$$ in Fig. 3). The Test Reuse workflow combines five main components:

1. Corpus of Documents is the set of documents that Word Embedding uses to build a Word Embedding Model.
2. Word Embedding creates a Word Embedding Model that encodes the semantic space of words and sentences, by mapping words and sentences to vectors that encode the semantic distance of corresponding elements in the Corpus of Documents.
3. Event Descriptor Extractor extracts the (textual) descriptors *D* of both the source event $e^s$ and the set of candidate target events $E^t =\{ e^t_0, e^t_1, \cdots e^t_n \}$ from the GUI state.
4. Semantic Matching Algorithm returns the set $E^t$ of elements sorted according to the similarity score of the descriptors of the target $\{D^t_0, D^t_1, \cdots D^t_n\}$ and source $D^s$ events.
5. Event Selector returns a test case $t^t=\langle e_0^t \ldots e_n^t \rangle$ for the target application $A^t$, where the event $e_i^t$ either matches an event $e_i^s$ in the test case $t^s$ of the source application $A^s$ or complete the sequence of events to obtain an executable test.

The glossary in Table 1 summarizes few core terms that we use in this paper.

**Table 1:** Glossary

test reuse approach	An approach that generates test cases for a target application by migrating test cases of a source application with semantic matching
semantic matching configurations	A four-tuple $\langle$Training Set, Word Embedding, Descriptor Extractor, Semantic Matching Algorithm$\rangle$ that defines the configuration for a semantic matching algorithm for instance $\langle$SemFinder, Intersection, WM, GooglePlay$\rangle$
semantic matcher	A component of Test Reuse that computes the semantic similarity of target events to a given source event
test generator	A component of Test Reuse that explores the target application to find a match for each event in $t_s$, and it generates a test $t_t$ for the target application
semantic matching evaluator	The framework that we use to evaluate semantic matching queries
fidelity plug-in	Measures the fidelity of the source and migrated test cases with respect to the ground truth
test migration evaluator	The framework that integrates the Semantic Matching Evaluator with both the ATM and CraftDroid Test Generator, as well as with a *Fidelity* plug-in
scenario	A test case $t^s$ of the source application with the ground truth $t^{gt}$, and a target application

### Corpus of Documents

The quality of a Word Embedding model depends on the Corpus of Documents used to train the model. There are two characteristics that the Corpus of Documents should have to obtain an effective Word Embedding model. The corpus shall both include as many distinct words as possible, as the model cannot compute similarity scores of words not represented in the vector space (Out-of-Vocabulary issue Bojanowski et al. 2017), and reflect the way mobile apps commonly use words. Indeed, a word can have a different meaning depending on the context of usage, and Word Embedding models trained with domain-specific corpora often outperform those trained with general corpora (Li et al. 2018).

We considered both general and mobile apps specific corpora, to study and quantify the importance of the context of usage. Our study considers three corpora of English documents that are available in our replication package:

**Blog Authorship Corpus (Blogs)** (Schler et al. 2006) that consists of 681,288 posts from 19,320 bloggers. This is a well-known corpus often used by the NLP and information science communities (Schwartz et al. 2013; Abbasi et al. 2008).

**User Manuals of** Android ** apps (Manuals)** (Behrang and Orso 2019) that consists of the user manuals of 500 Android applications. This corpus was built by the authors of ATM (Behrang and Orso 2019), who used it to train a Word2vec  Word Embedding model for running ATM.

**Apps Descriptions (Google-play)** that consists of the English descriptions of 900,805 Android apps in the Google Play Store. We constructed this corpus by crawling the list of similar apps of each crawled page. We used as seeds of the crawler the pages of the apps returned by searching random words in the Google Play search bar. We stopped crawling Google Play when the crawler could not find any new application in 24 hours.

### Word Embedding

Word Embedding (Mikolov et al. 2013a) is a class of unsupervised language modeling and feature learning techniques that map words and sentences from a Corpus of Documents to vectors of real numbers (Turian et al. 2010).

A Word Embedding model assigns each word in the corpus to a unique vector in the space. Words that share common contexts in the corpus are close in the space. Test Reuse approaches use Word Embedding models to identify semantically similar, although syntactically different words that independent developers may use to name actions with similar semantics. We experimented with the Word Embedding techniques that are most commonly used in software engineering (Jiao and Zhang 2021).

**Word2Vec (**W2V**)** Mikolov et al. (2013a): one of the most popular Word Embedding techniques developed in 2013 in Google. It implements a two-layer neural network that is trained to reconstruct linguistic contexts of words.

**Global Vectors (**GloVe**)** Pennington et al. (2014): a probabilistic technique that learns vectors or words from their co-occurrence information (how frequently they appear together in the corpus).

**Word Mover’s distance (**WM**)** Kusner et al. (2015): a Word Embedding technique based on the observation that semantic relationships are often preserved in vector operations on Word2vec models. For instance, vector(London) - vector(England) + vector(Germany) is close to vector(Berlin). WM exploits this property by finding the minimum *traveling distance* between strings (Kusner et al. 2015). As such, WM considers distance between strings (one or more words) (Turian et al. 2010) and not only among pairs of words like the distances based on Word2vec or GloVe (Turian et al. 2010). In the context of Test Reuse this could be useful, because event descriptors often contain multiple words (Behrang and Orso 2019; Lin et al. 2019). WM returns an integer greater than zero, that we normalize from 0 to 1, with a standard normalization $1/{(1 + \text {WM}(\text {txt}^s, txt ^t) )}$.

**FastText (**Fast**)** (Bojanowski et al. 2017): an extension of Word2vec developed in Facebook. While Word2vec treats words as the smallest unit to train on, FastText learns vectors for the n-grams that are found within each word. FastText computes the vector of a word as the sum of its n-grams. For example, the word “aquarium” has the n-grams: “aqu/qua/uar/ari/riu/ium”. FastText is designed to alleviate the Out-of-Vocabulary issue (Bojanowski et al. 2017). In fact, even if the word “aquarius” is not present in the corpus, FastText would embed “aquarius” near to "aquarium" because they share five n-grams.

**Bidirectional Encoder Representations from Transformers (**BERT**)** (Devlin et al. 2018): a context-sensitive Word Embedding technique that infers the meaning of words from its surroundings, by training a model on 15% of masked words in sentences. While directional models read the text input sequentially either left-to-right or right-to-left, BERT reads the entire sequence of words at once, thus allowing the model to learn the context of a word in its left and right surrounding.

**Neural Network Language Model (**NNLM**)** (Arisoy et al. 2012): a family of neural network techniques that learn Word Embedding models jointly with a language model. The model is expressed as a function that captures the distribution of sequences of words in a natural language. The language model estimates the probability of words occurring after a prefix. Thus NNLM are context-sensitive.

In our study we consider the NNLM technique proposed by Hub (2020).

**Universal Sentence Encoder (**USE**)** (Cer et al. 2018): a context-sensitive Word Embedding technique that Google proposes in two variants, *Transformer*-based and *Deep Averaging Network*-based, that privilege accuracy and consumption of computing resources, respectively. We used the *Deep Averaging Network* variant in our study.

Both ATM and CraftDroid rely on models built with Word2vec, AdaptDroid works with Word Movers Distance.

### Event Descriptor Extractor

The Event Descriptor Extractor gets the descriptors of the events that Semantic Matching Algorithm needs to compute the similarity among the source $e^s$ and candidate target events $\langle e^t_0, e^t_1, \cdots e^t_n,\rangle$.

An event descriptor *D* is a set of textual *attributes* $\{ a_1, a_2 \cdots a_m \}$ in the GUI states.

Each attribute is a $\langle type, value\rangle$ pair, for instance $\langle text,press~ok\rangle$. Our study considers all the attribute types used in ATM, CraftDroid, and AdaptDroid (Lin et al. 2019; Behrang and Orso 2019; Mariani et al. 2021). All approaches consider both *primitive* and *derived* attributes. *Primitive* attributes are directly associated with the widget of the event under consideration. *Derived* attributes are attributes that are not directly associated with the widget itself, but to some near widgets (Becce et al. 2012). For instance in the second window of Fig. 1 (A), the attributes of widget $e^s_2$ are empty, the corresponding field in the window is blank, and we infer the semantics of the widget from the text attribute “Price” of a neighbor widget in the same window.

The *primitive* attributes of a widget *w* are:

**text**, the visible label associated with *w* (xml attribute android:text).

**content-description**, a textual description of *w* that is not visible in the GUI. It is often used by Android Accessibility APIs as alternate text for describing the widget to visually impaired users (xml attribute android:contentDescription).

**hint**, a textual description of *w* that is used in editable widgets to help the user to fill the correct content (xml attribute android:hint).

**resource-id**, the unique identifier of *w* that developers assign to each widget to reference them in the code (xml attribute android:id).

**file-name**, the name of the file associated with *w*. For example, the name of the image file associated with a widget.

**activity-name**, the name of the Android activity of the widget *w*.

Both ATM and AdaptDroid define derived attributes from the spatial positions of the widgets (Behrang and Orso 2019; Mariani et al. 2021). CraftDroid defines derived attributes from the hierarchical structure of the Android GUI states (Lin et al. 2019), in which widgets have a parent-child-sibling relationship. The *parent* element directly precedes the *child* element in the hierarchy, and *siblings* elements share the same parent.

The derived attributes of a widget *w* are:

**parent-text**, the *text attribute* of the parent widget of *w*.

**sibling-text**, the *text attribute* of the sibling widget before *w* in the widget hierarchy.

**neighbor-text**, the *text attribute* of the widget closest to *w* in the page, within a given distance. If there are no widgets within the given distance or the attribute of the closest widget is empty, the value of the derived neighbor-text is also empty.

Some *attributes* of a widget can be undefined (empty). For example, most widgets lack the *hint* or *content-desc attributes*.

In our experiments we consider the groups of *attributes* of ATM (“A” in Table 2), CraftDroid (“C” in the table), their intersection (A $\cap$ C) and union (A $\cup$ C). The *attributes* of AdaptDroid are the same of ATM, except for *hint* that ATM considers, and AdaptDroid does not, and that is empty in our case studies. These groups allow us to evaluate also the impact of sets attributes that are used by an approach only. For example, we can evaluate the impact of the descriptors *neighbor-text* and *file-name*, by comparing the results of groups “A” and “A $\cap$ C”.

**Table 2:** Groups of event descriptors

Attribute	Attribute	ATM	Craftdroid	Intersection	Union
category	type	A	C	A $\cap$ C	A $\cup$ C
Primitive	Text	✓	✓	✓	✓
	Resource-id	✓	✓	✓	✓
	Content-desc	✓	✓	✓	✓
	Hint	✓	✓	✓	✓
	File-name	✓			✓
	Activity-name		✓		✓
Derived	Neighbor-text	✓			✓
	Parent-text		✓		✓
	Sibling-text		✓		✓

**Algorithm 1:**
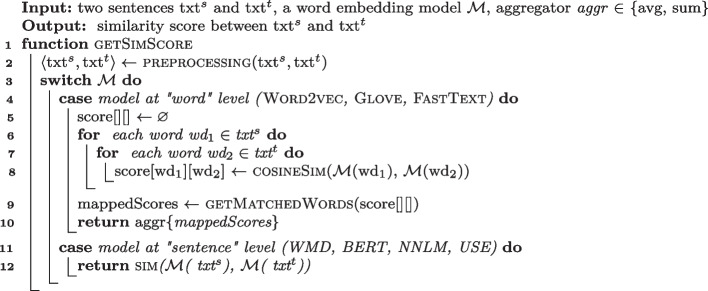
Semantic similarity calculator.

### Semantic Matching Algorithm

Semantic Matching Algorithm returns the set of candidate target events $E^t$ that correspond to a source event $e^s$, sorted according to the similarity scores between $D^s$ and $D^t_i$, where $D^s$ is the descriptor of the source event $e^s$, and $D^t_i$ are the descriptors of the event $e^t_i \in E^t$.

In our study we consider the Semantic Matching Algorithm of ATM, Craft-Droid, AdaptDroid, as well as SemFinder, a novel Semantic Matching Algo-rithm that we propose in this paper. All the algorithms compute the semantic similarity scores among the attribute values of the source and target descriptors using a Word Embedding model $\mathcal {M}$. All algorithms compute the semantic similarity of values of the textual attributes of the source and target descriptors, $txt ^s$ and $txt ^t$, with Algorithm 1 (Function getSimScore). Function getSimScore computes a real number that expresses the similarity score between $txt ^s$ and $txt ^t$. Function getSimScore first pre-processes the strings by removing stop words, lemmatizing the strings, and splitting words originally in camel case notation (Line 2). It then scores the similarity of the pre-preprocessed strings with respect to either a word (Word2vec, GloVe, FastText) or a sentence (*WM, BERT, NNLM, USE*) level model $\mathcal {M}$.

**Table 3:** Synopsis of the semantic matching algorithms

Semantic matching algorithm	Type of considered attributes	Similarity score aggregation of attributes	Similarity score aggregation of words
ATM	Prioritized set	Sum	Max
CraftDroid	Same type	Mean	Average
AdaptDroid	Prioritized set	N/A	Average
SemFinder	All	N/A	Average

**Word Level Models** Function getSimScore computes the cosine similarity of vector($wd_1$) and vector($wd_2$) for all possible pairs of words of the two strings $\langle wd_1 \in txt ^s, wd_2 \in txt ^t\rangle$ (Lines 6–8). Then it identifies the best match among the pairs as the pair with the highest cosine similarity, where every word is matched only once (Line 9). It finally returns the similarity score computed with the input aggregation function (Line 10). ATM aggregates by summing the scores (*sum*), while CraftDroid, AdaptDroid, and SemFinder aggregate by averaging the scores (*avg*).

**Sentence Level Models** Function getSimScore queries the model $\mathcal {M}$ with the strings as a whole. Notably, both ATM and CraftDroid use models at word level, we add Lines 10 and 12 to make the algorithm compatible with the sentence level Word Embedding models that we considered in our study.

Table 3 summarizes the key differences among the four algorithms that we described in details. SemFinder scores the similarity of the pre-preprocessed strings with respect to sentences (value *N/A* in column *Similarity score aggregation of attributes*) and aggregates words by average, like AdaptDroid, while ATM and CraftDroid score the similarity of the pre-preprocessed strings with respect to words and aggregate attributes by sum and max, and words by max and average, respectively (columns *Similarity score aggregation of attributes* and *Similarity score aggregation of words*). ATM and AdaptDroid select the type of considered attributes between source and target app by priority, while CraftDroid considers only attributes of the same type in both source and target app, and SemFinder considers all attributes that are in the target app (column *Type of considered attributes*). The results that we discuss in Section 4 show that widening the set of considered attributes as in SemFinder enhances the chances of finding a corresponding event, and improves over previous approaches when combined with the words scored with respect to sentences and aggregated by average as in both AdaptDroid and SemFinder.

**Algorithm 2:**
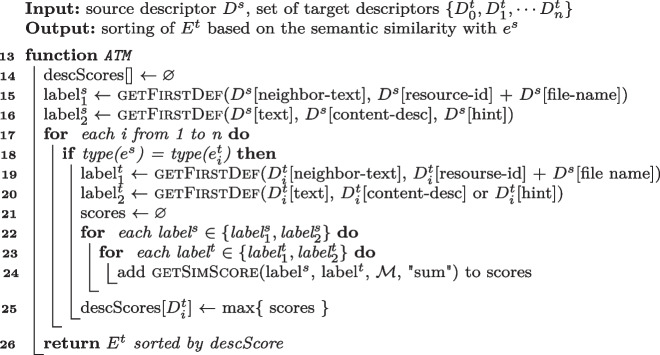
Semantic matching algorithm of ATM.

**Semantic Matching of ATM**  (Behrang and Orso 2019) Lines 13 to 26 of Algorithm 2 encode the Semantic Matching Algorithm of ATM. The algorithm collects two textual representations of the source event: *label*$^s_1$ (Line 15) and *label*$^s_2$ (Line 16). The algorithm initializes *label*$^s_1$ to the first *defined* attribute among $\langle$*neighbor-text*, *resource-id* $+$ *file-name*$\rangle$ in $D^s$. If all of such attributes are undefined, the algorithm initializes *label*$^s_1$ to the empty string. ATM extracts the *neighbor-text* attribute only for filling events, while for clicking events it considers the attribute as undefined. The algorithm initializes *label*$^s_2$ to the first *defined* attribute among $\langle$*text*, *content-desc*, *hint*$\rangle$ in $D^s$ (Line 16). For each event $e_i^t \in E^t$ that has the same type of $e_s$ (either both filling or both clicking events), the algorithm collects *label*$^t_1$ and *label*$^t_2$ in the same way it collects *label*$^s_1$ and *label*$^s_2$, respectively. Then, the algorithm invokes Function getSimScore (Algorithm 1) for each combination of $\langle$*label*$^s \in \{label _1^s, label _2^s\}$, *label*$^t \in \{label _1^t, label _2^t\} \rangle$, using "sum" as aggregation function. The algorithm assigns the highest returned value to the score of the current target event (*score*[$D^t_i$] Line 25), and sorts $E^t$ based on the final scores (Line 26).

**Algorithm 3:**
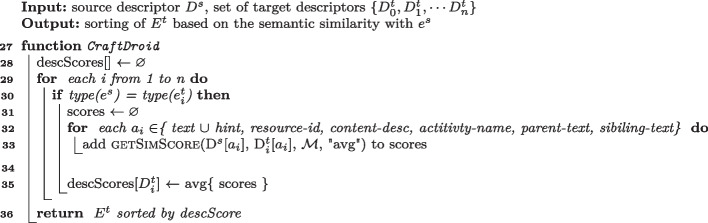
Semantic matching algorithm of CraftDroid.

**Semantic Matching of** CraftDroid  (Lin et al. 2019) Lines 27 to 36 of Algorithm 3 encode the Semantic Matching Algorithm of CraftDroid. For each target event $e^t_i$ of the same type of $e^s$ CraftDroid gets the similarity scores of their descriptor attributes (Line 32) and adds them to List *scores*. The algorithm only compares corresponding attributes. For example, it compares *resource-id* of the source descriptor only to *resource-id* of the target descriptor. CraftDroid computes the final score of the current target descriptor as the average of List *scores* (Line 35).

**Algorithm 4:**
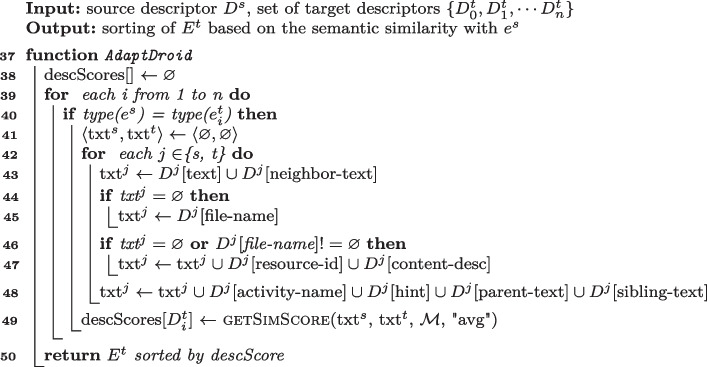
Semantic matching algorithm of AdaptDroid.

**Semantic Matching of** AdaptDroid  (Mariani et al. 2021) Lines 37 to 50 of Algorithm 4 encode the Semantic Matching Algorithm of AdaptDroid. For each event $e_i^t \in E^t$ that has the same type of $e_s$ AdaptDroid builds two strings *txt*$^s$ and *txt*$^t$, by concatenating the values of the attributes of $D^s$ (separated by a white space) and $D^t$ as follows: (step i) It adds $\langle$*text*, *neighbor*$\rangle$ in $D^s$ to *txt*$^s$ (Line 43);(step ii) If *txt*$^s$ is still empty, it adds $\langle$*file-name*$\rangle$ to the *txt*$^s$ (Line 45); (step iii) If either *txt*$^s$ remains empty or there is an associated file (*file-name* not empty), it adds $\langle$*resource-id*, *content-desc*$\rangle$ to the *txt*$^s$ (Line 47); (step iv) It adds $\langle$*activity-name*, *hint*, *sibling-text*, *parent-text*$\rangle$ (Line 48). It computes the score of the current target descriptor by averaging the similarity scores between *txt*$^s$ and *txt*$^t$ (Line 49). AdaptDroid Semantic Matching Algorithm balances the effect of too many attributes that may lead to a noisy text and too few attributes that may lead to insufficient semantic information, by adding attributes in a specific order, only if necessary. For example, a non empty $\langle$*file-name*$\rangle$ attribute usually indicates the presence of an *ImageButton*, and consequently the need of additional textual information to get an accurate semantics.

**Algorithm 5:**
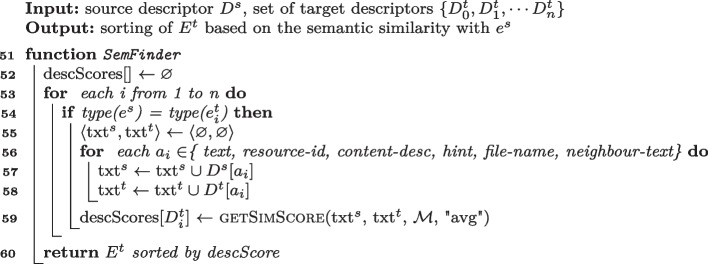
Semantic matching algorithms of SemFinder.

**Semantic Matching of** SemFinder Lines 51 to 60 of Algorithm 5 encode the Semantic Matching Algorithm SemFinder that we propose in this paper. For each event $e_i^t \in E^t$ that shares the type of $e_s$ SemFinder builds two strings *txt*$^s$ and *txt*$^t$. It (i) builds txt$^s$ by concatenating all the values of the attributes of $D^s$ (separated with a space), (ii) builds *txt*$^t$ with the values in $D^t_i$, (iii) prunes words repeated in the same string, (iv) aggregates the similarity score between *txt*$^s$ and *txt*$^t$ using *average*, and (v) assigns the result to the final score of the current target descriptor (Line 59). The SemFinder intuition is that even though some attributes could be sometime more important than others, strict prioritization could result information loss. While collecting the values of a fixed subset of attributes and concatenating them without prioritization could be a safer option.

ATM, CraftDroid, AdaptDroid, and SemFinder share the general framework, and differ in three main aspects: the type of attributes they consider, as we discuss in Section 3.3, the way they aggregate the similarity scores of multiple pairs of attributes, and the way they aggregate the similarity scores of word-level models.

**Fig. 4:**
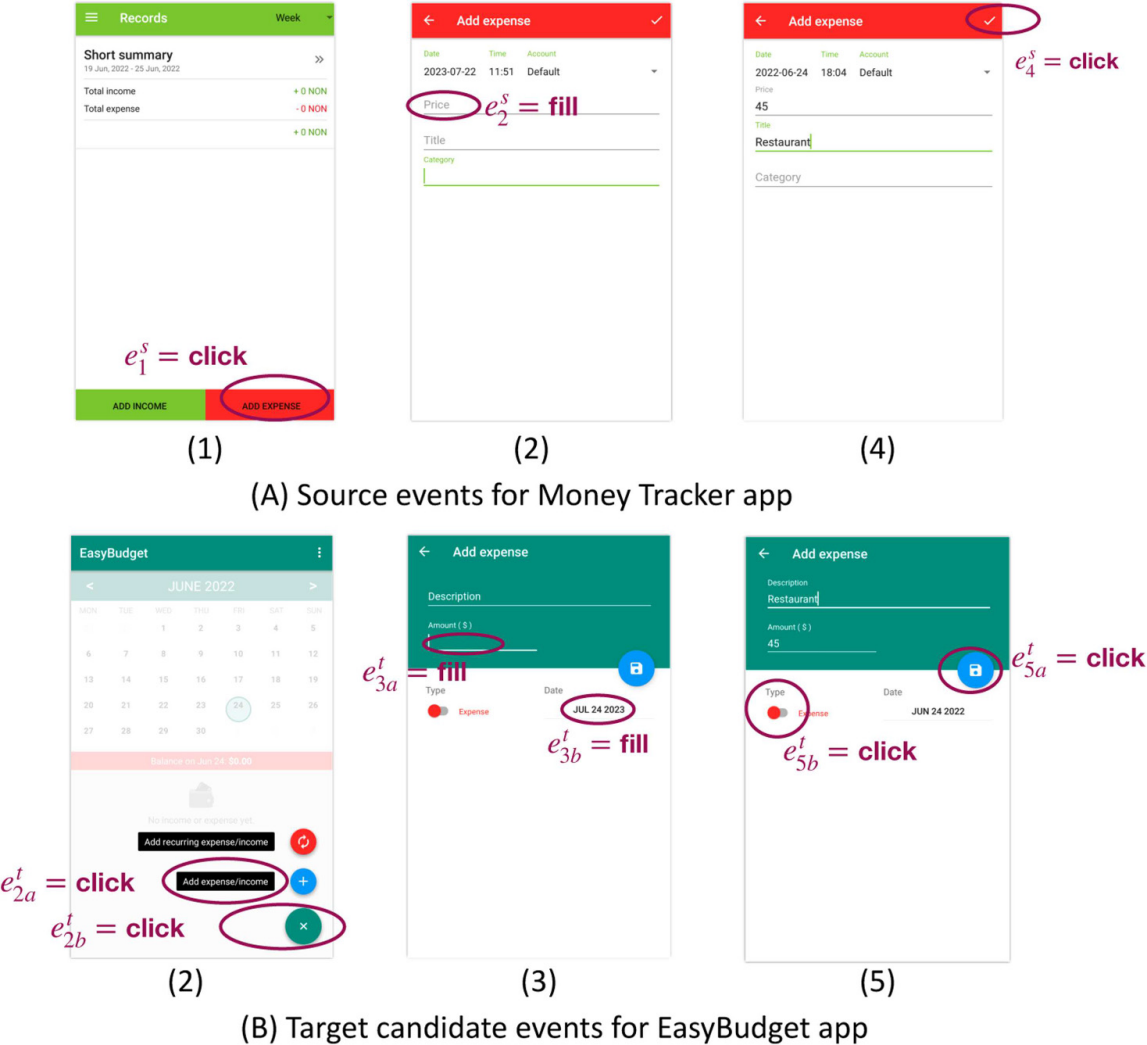
Excerpt of test reuse example, the target test cases (B) is obtained by migrating the source test case (A)

We illustrate how ATM, CraftDroid, AdaptDroid and SemFinder differ with the three pairs of matching events of Fig. 4 that we excerpt from Fig. 1. Figure 4 reports three events of the source test case ($e^s_1$, $e^s_2$ and $e^s_4$) in the corresponding windows, and three pairs of target candidate events in the corresponding windows ($e^t_{2a}$ and $e^t_{2b}$, $e^t_{3a}$ and $e^t_{3b}$, $e^t_{5a}$ and $e^t_{5b}$), being $e^t_{xa}$ the correct candidate. All approaches consider a possibly large number of candidate events. In this example, we limit the number of candidate events for each source event to two events.

**Type of Considered Attributes of Source and Target Descriptors** The algorithms compare attributes by considering either specific combinations of types of source and target attributes or a priority between attributes. CraftDroid selects attributes by type only, and compares only attributes with the same type. ATM selects attributes by both type and priority, and compares attributes of a subset of combinations of types, and prioritizes attributes according to their order, by considering the first not-empty attribute. AdaptDroid selects attributes by priority only, according to the types of the attributes. SemFinder compares the set of attributes as a whole.

**Table 4:** Selection of attributes for event $e^s_2$ with each Semantic Matching Algorithm

Source$^{a}$ Event	Algorithm	Target$^{b}$ Event	Pairs for similarity score	Aggregtion	Score$^{c}$
$e^s_2$	CraftDroid	$e^t_{3a}$	No pairs	average	0
		$e^t_{3b}$	No pairs	average	0
	ATM	$e^t_{3a}$	$\langle label ^s_2$:price, $label ^t_1$:amount$\rangle$= 0.58, other pairs get score of 0	max	0.58
		$e^t_{3b}$	$\langle label ^s_2$:price, $label ^t_1$:date$\rangle$= 0.61, other pairs get score of 0	max	0.61
	AdaptDroid	$e^t_{3a}$	$\langle txt ^s$:price, $txt ^t$:amount$\rangle$= 0.58	NA	0.58
		$e^t_{3b}$	$\langle txt ^s$:price, $txt ^t$:date$\rangle$= 0.61	NA	0.61
	SemFinder	$e^t_{3a}$	$\langle txt ^s$:price, $txt ^s$:amount cost$\rangle$= 0.67 s	NA	0.67
		$e^t_{3b}$	$\langle txt^s$:price, *txt*$^t$:date calendar$\rangle$ = 0.61	NA	0.61

$^{a}$ The descriptor of $e^s_2$ is $D^s=[$ *text*: price]

$^{b}$ The descriptor of $e^t_{3a}$ and $e^t_{3b}$ are $D_{3a}^t = [$*neighbor*: amount, *id*: cost] and $D_{3b}^t = [$*neighbor*: date, *id*: calendar], respectively

$^{c}$ Semantic Matching Algorithms used Word2vec pre-trained model to calculate similarity scores

Table 4 summarizes how the Semantic Matching Algorithms calculate the similarity score of the two target events, $$e^t_{3a}$$ and $$e^t_{3b}$$ with respect to the event $$e^s_2$$, by using the Word2vec model trained on Manuals corpus. The footnotes in the table indicate the descriptors of the events, $$D^s=[ text : $$ price] for $$e^t_{3a}$$, $$D_{3a}^t = [$$*neighbor*: amount, *id*: cost] for $$e^t_{3a}$$, $$D_{3b}^t = [$$*neighbor*: date, *id*: calendar] for $$e^t_{3b}$$. The table reports the pairs of attributes that each algorithm considers for similarity score with the individual scores for the pairs of attributes (column *Pairs for similarity score*), the aggregation function (column *Aggregation*) and the computed score for the pairs of events (column *Score*).

CraftDroid does not compare any pairs of attributes, since source and target events have different type of attributes, thus it scores zero for both target events.

ATM computes a similarity greater than zero only for a pair of labels for each of the two pairs of events: $$\langle label ^s_2:price, label ^t_1:amount\rangle = 0.58$$ for $$\langle e^s_2, e^t_{3a} \rangle $$, $$\langle label ^s_2:price, label ^t_1:date\rangle = 0.61$$ for $$\langle e^s_2, e^t_{3b} \rangle $$.^2^. ATM scores zero for all other pairs of labels, since they contain at least an empty label: * neighbor-text, resource-id*, *file-name* attributes for $$ label ^s_1$$, * text, content-desc*, *hint* for $$ label ^t_2$$.

In the last step, ATM uses *max* aggregation function and chooses the only non-zero combination of labels as the final score.

AdaptDroid combines the attributes with highest priority of each event into a text *txt*, and scores only the resulting pairs of text: $\langle txt ^s:price, txt ^t:amount\rangle = 0.58$ and $\langle txt ^s:price, txt ^t:date\rangle = 0.61$.

SemFinder also combines the attributes of each event in text, but it considers all attributes, differently from AdaptDroid. In this example, SemFinder produces different texts for both target events that are associated with the two attributes. Consequently the final score is also different.

In this example, SemFinder is the only Semantic Matching Algorithm that computes the highest score for the correct candidate $e^t_{3b}$.

**Similarity Score Aggregation of Multiple Pairs of Attributes** ATM and CraftDroid aggregate similarity score of attributes, while AdaptDroid and SemFinder do not, since they combine the attributes in a single *txt*. ATM aggregates the similarity score of multiple pairs of attribute types by *max*imum (Line 25 of Algorithm 2), while CraftDroid aggregates them by *average* (Line 34 of Algorithm 3). ATM aggregation by maximum misses pairs of attributes that may be relevant but with low score while CraftDroid.s aggregation by average does not.

In the running example of Fig. 4, the source event $e^s_1$ has the descriptor $D^s_1$= [*resource-id*: “button”, *content-desc*: “expense”], and the two target events $e^t_{2a}$ and $e^t_{2b}$ have the descriptors $D^t_{2a}=$[*resource-id*: “button”, *content-desc*: “expense”] and $D^t_{2b}$=[*resource-id*: “button”, *content-desc*: "exit"], respectively. ATM and CraftDroid computes the score of four pairs of attributes, two for each pair of events. In both cases three of the four pairs of attribute score 1, $\langle expense, expense\rangle$ and $\langle button, button\rangle$, twice, thus both pairs of events score 1. With the aggregation by maximum (ATM), both $\langle e^s_1, e^t_{2a}\rangle$ and $\langle e^s_1, e^t_{2b}\rangle$ score 1, thus it does not provide useful information to chose the target event. With the aggregation by average (CraftDroid), $\langle e^s_1, e^t_{2a}\rangle$ scores 1 and $\langle e^s_1, e^t_{2b}\rangle$ scores 0.56, thus it identifies the correct matching.

**Similarity Scores Aggregation of Word Embedding Models** ATM aggregates the similarity scores of words in a string by summing the similarity scores of words (Line 24 of Algorithm 2). CraftDroid aggregates the scores of words by *average* (Line 33 and 49 of Algorithm 3). Both AdaptDroid and SemFinder use *average* as well. Aggregation by *sum* privileges (assign high score to) strings with many words, and may assign a higher score to two attributes with many unrelated words than to two attributes with fewer highly related (semantically similar) words, as the semantic score of two words in the model is always positive.

In the running example of  Fig. 4,  the source event $$e^s_4$$ has the descriptor $$D^s_4=[resource-id :\text {``save~expense~entry''}]$$, and the two candidate events $$e^t_{5a}$$ and $$ e^t_{5b}$$ have the descriptors $$D_{5a}^t$$ = [*resource-id:* set expense"] and $$D_{5b}^t~=~$$[*resource-id*: set expense type"], respectively. The Word2vec model trained with Manuals computes a score for the three pairs from the three words that occur in the attribute of the source event combined with the three words that occur in the attributes target events as: $$\langle expense, expense \rangle =1$$, $$\langle save, set\rangle = 0.66$$, $$\langle entry, type\rangle =0.65$$. CraftDroid aggregates the scores by average: $$score(e^t_{5a}) = 0.83$$, $$score(e^t_{5b}) = 0.73$$, while ATM aggregates by sum $$score(e^t_{5a}) = 1.66$$, $$score(e^t_{5b}) = 2.31$$. In this case, CraftDroid assigns higher score to the correct candidate event, while ATM does not.

### Event Selector

The Event Selector builds a test case $t^t$ for the target application $A^t$ by chaining the candidate events that the Semantic Matching Algorithm suggests as semantic matches of the events in the source test case $t^s$. The Event Selector incrementally processes the events $e_i^s$ of the source test case $t^s$. It retrieves a set $E^t$ of candidate events that correspond to the current event $e_i^s$ from both the current state of the target application $A^t$ and the Target Application Model. The Target Application Model is the GUI model used in ATM, CraftDroid and AdaptDroid. It is a directed graph where nodes correspond to the states of the GUI, and edges are labelled with the events that lead from the state that corresponds to the source to the state that corresponds to the target node of the edge, respectively.

ATM retrieves the candidate events from the current state of the app under test, and considers the events in the Target Application Model only if the Semantic Matcher does not find any event in the current state with a semantic similarity score above a constant threshold.

CraftDroid retrieves the candidate events from both the current state and the Target Application Model. AdaptDroid retrieves events from the current state, and selects the candidate events as the events with a semantic similarity score above a constant threshold. If AdaptDroid does not find events with a semantic similarity score above the threshold in the current state, the Event Selector looks for an event that belongs to a state different from the current state, and it selects a sequence of events that head to the candidate event, from the Target Application Model (*ancillary events* in Fruiter Zhao et al. 2020, *leading events* in CraftDroid Lin et al. 2019.)

Once identified a matching event $e^t$ for $e_i^s$, Event Selector adds the event (and the leading events, if any) to the test case for the target application $t^t$, and computes the next current state of the target application $A^t$ by executing the added events. When ATM and CraftDroid do not find a matching event, they skip $e_i^s$ and proceed with $e_{i+1}^s$, while AdaptDroid randomly selects an event as next candidate. AdaptDroid improves the migrated test cases $t^t$ with a genetic algorithm that uses the test cases as the initial population and the Target Application Model to repair infeasible tests that the crossover operations generate during the evolution. In our study we considered the implementations of Event Selector of both CraftDroid and ATM. We excluded the Event Selector of AdaptDroid from our study as its genetic algorithm is too computationally expensive, which would have drastically limited the scale of our experiments. Nevertheless, CraftDroid and ATM are two state-of-the-art approaches, which are representative of Test Reuse for Android applications.

#### ATM Event Selector

Lines 62 to 83 of Algorithm 7 encode the ATM Event Selector algorithm that initializes a Target Application Model (Line  63), and iteratively looks for next events that match the input source events, until either a matching event is found or a timeout expires (call Function findNextEvent at Line 69).

Function findNextEvent selects the next event that corresponds to $e^s_i$ (Line 84) by (i) looking for a matching event in the current state (Lines 88-95), (ii) looking for a matching event in the Target Application Model (WTG in ATM terminology), if it does not find a matching event in the current state and the source event is a GUI event (and not an oracle event) (Lines 96-103), (iii) randomly selecting an event in the current state, if it does not find a matching event in the Target Application Model (Lines 105-107), (iv) moving back to the former page, after a maximum number of randomly selected events (Lines 108-110).

It computes the next current state by executing the events that findNextEvent selects (Line 70), adds the events to a buffer (Line 70), and updates both the Target Application Model (Line 71) and the target test case $t^t$ (Lines 73-76). It updates the target test case $t^t$ by adding the events (Line 76) after simple syntactic checks if the events are oracle events (Line 74). If Event Selector cannot find a matching event within a timeout, it rolls back to the state of previous matched event, and restarts from an alternative event.

**Algorithm 6:**
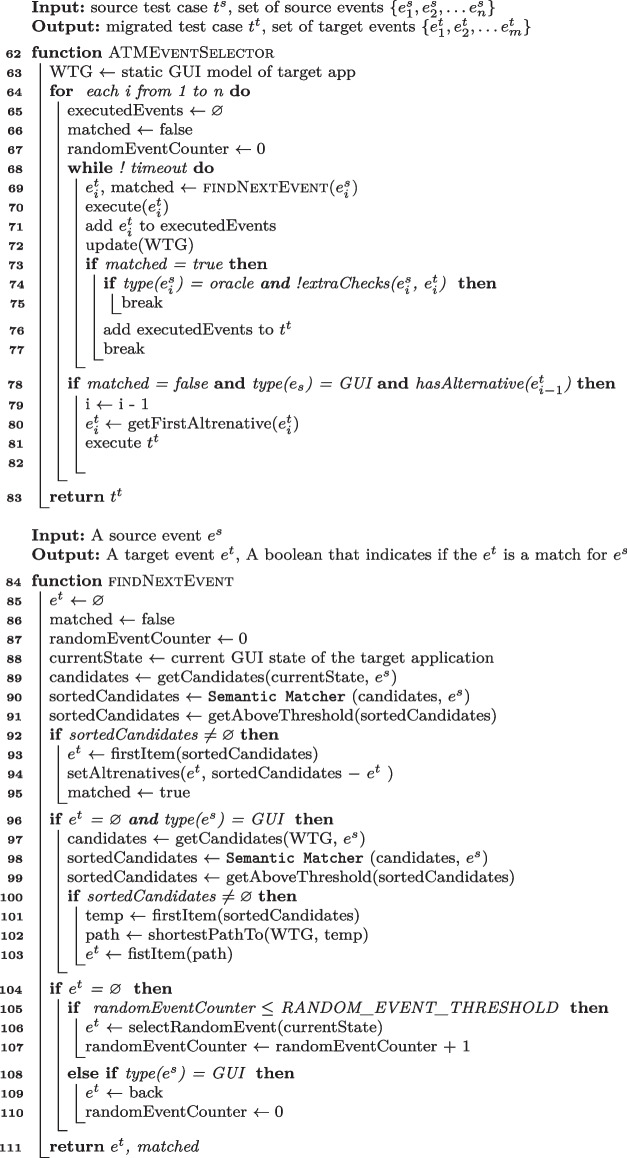
ATM Event Selector Algorithm.

#### CraftDroid Event Selector

Lines 110 to 129 of Algorithm 7 encode the CraftDroid Event Selector algorithm. CraftDroid initializes the Target Application Model (UITG in CraftDroid terminology) (Line 111), and iteratively generates test cases that correspond to the input source test case $t^s$ (Lines 112-129), aiming to maximize a test similarity score, computed as the average similarity score of the events in the test. It terminates either when the test similarity score does not improve (Line 129) or when a timeout expires.

It generates the tests by scanning the events $e^s_i$ in the input source test case $t^s$, and looking for events that match the current event in $t^s$. It selects all GUI events in both the current state and the Target Application Model (Line 116) and all oracle events in the current state only (Line 119), and sorts them by semantic similarity with respect to the current event $e_i^s$ in the source test cases $t^s$ (Line 120).

It computes the leading events for the candidate events (at the top of the *sortedCandidate* list), that is, the sequences of events that lead to a state in which the event is executable (call to getLeadingEvents at Line 122), and adds the non-empty leading event sequence to the target test case $t^t$ (Line 126), after few simple syntactic checks for oracle events (Line 124).

Function getLeadingEvents retrieves all paths in the Target Application Model that lead to the event $e^t$ (Line 132), and incrementally executes them starting from the shortest ones, till it finds an executable sequence of leading events to return (Lines 133-137).

**Algorithm 7:**
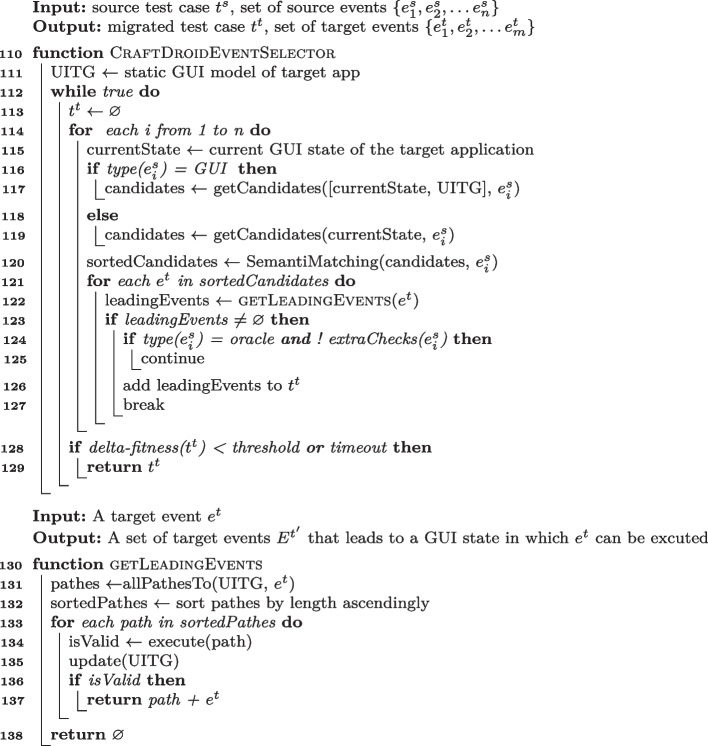
CraftDroid Event Selector Algorithm.

## Experiment

We evaluate semantic matching both in isolation and in the context of Test Reuse. The evaluation in isolation (research questions RQ1, RQ2, RQ3) investigates the effectiveness of semantic matching for a broad set of configurations in controlled scenarios, without referring to a specific Test Reuse approach. The evaluation in the context of Test Reuse (research questions RQ4, RQ5, RQ6) investigates the effectiveness and impact of semantic matching when used with the test generation process. We empirically evaluated semantic matching in isolation and in the context of Test Reuse with 95 and 89 pairs of source and target test cases $\langle t_s, t_t \rangle$ from the test migration scenarios provided by ATM and CraftDroid for 30 apps, respectively.

### Semantic Matching in Isolation

- **RQ1** **Baseline Comparison:** *Do semantic approaches based on word embedding outperform syntactic and random approaches?*
- **RQ2** **Component Effectiveness in Isolation:** *What are the most effective instances of each component on the semantic matching of GUI events?*
- **RQ3** **Component Impact Analysis in Isolation:** *Which component types have the greatest impact on the semantic matching of GUI events?*

RQ1 validates the usefulness of semantic matching for Test Reuse by comparing the effectiveness of semantic approaches to both syntactic (Edit Distance Similarity and Jaccard Similarity) and random approaches. Syntactic and random approaches are agnostic to the semantics of textual information, thus the comparison with these approaches well indicates the contribution of the semantic approaches. RQ2 studies the implementations of different components of semantic matching approaches, and identifies the implementations that perform best. RQ3 studies the impact of the component types on the effectiveness of semantic matching, and identifies the most impactful ones. We report the results of experimenting with 337 different configurations in controlled scenarios.

### Semantic matching in the context of test reuse

 

- **RQ4** **Impact of Semantic Matching in the Context of Test Reuse:** *Does the effectiveness of semantic matching impact on test reuse?*
- **RQ5** **Component Effectiveness in the Context of Test Reuse:** *What are the most effective instances of each component for test reuse?*
- **RQ6** **Component Impact Analysis in the Context of Test Reuse:** *Which component types have the most relevant impact on test reuse?*

RQ4 studies the correlation between semantic matching and Test Reuse, and identifies the combinations of configurations that achieve the most effective Test Reuse. RQ5 studies the impact of the implementations of different components on Test Reuse, and identifies the implementations with the best impact on Test Reuse. RQ6 studies the impact of the component types on Test Reuse, and identifies the critical component types. We report the results of experimenting semantic matching with two state-of-the-art Test Reuse approaches: ATM and CraftDroid.

### Subjects

For our experiments, we considered all publicly available test migration scenarios of both ATM and CraftDroid: 248 scenarios, from 42 Android apps. Each scenario is a test case $t^s$ of the source application with the ground truth $t^{gt}$ (i.e., the migrated version of $t^s$) of a target application. We considered the 147 scenarios of the 30 Android apps that we could compile and execute. We could not experiment with 12 apps, since some of ATM apps are available in new versions that do not compile any more with the ATM scenarios, and some CraftDroid apps both require communication with a server and are available with new API or Security protocol not compatible with the CraftDroid scenarios. We pruned 52 redundant scenarios, that is, scenarios that occur in other scenarios, and experimented with the 95 unique and compatible scenarios. We addressed RQ1, RQ2 and RQ3 with experiments on all the 95 scenarios. We addressed RQ4, RQ5 and RQ6 with experiments on 89 scenarios for 29 out of 30 Android apps, since the FirefoxFocus app includes a key widget with an unconventional type incompatible with Appium, the test automation tool that we used in the experiments with the Test Reuse tools. We experimented CraftDroid on all scenarios, and ATM on ATM scenarios only, because ATM instruments the source code of the apps, and the instrumentation logic designed in the tool works only for the scenarios in the original study. Instrumenting different apps results in compilation errors. Thus, we identify two sets of scenarios:

- *Shared Scenarios*: 27 scenarios that both ATM and CraftDroid can process. We refer to these scenarios to comparatively evaluate semantic matching in the context of the two approaches applied to the same scenarios.
- *All Scenarios*: All 89 scenarios. We refer to these scenarios to investigate semantic matching with a wide range of applications.

Table 5 summarizes the scenarios we use in our experiments.

**Table 5:** Subjects of our experiment

Subject from	Category	Test case description	App name	# of DL$^{a}$
ATM	Expense Tracker	Add an expense entry to expense list	EasyBudget (Letondor 2021)	100k
			Expenses (Ferreira 2021)	1K
			Daily Budget (Kvannli 2021)	50K
			Open Money (xorum 2021)	1K
	Note Taking	Add a note and save	Swiftnotes (Chifor 2021)	−
			Writely Pro (plafu 2021)	−
			Pocket Note (roxrook 2021)	−
	Shopping List	Add a shopping item	Shop.List1 (Grzyb 2021)	−
			Shop.List2 (Vansuita 2021)	100K
			Shop.List3 (SECUSO Research Group 2021)	5K
			OI Shop. List (OpenIntents 2021)	1M
CraftDroid	Browser		Lightning (Restaino 2021)	10K
		Go to an URL, go to another URL	Privacy (Stoutner 2021)	1K
		go back to the first URL	FOSS (Gaukler 2021)	−
			FirefoxFocus (Mozilla 2021)	5M
	To-Do List		Minimal (Roy 2021)	−
		Add a todo task and save	Clear List (douzifly 2021)	−
		Remove the recent task		
			Todo List (SECUSO Research Group 2021)	−
			Simply Do (Kildare 2021)	−
			Shop. List (Kildare 2021)	−
	Shopping	1) Sign up	Rainbow (rainbowshops 2021)	0.5M
		2) Sign in		
			Yelp (Yelp Inc 2021)	50M
	Mail Client	1) Search for an email	Mail.ru (Mail.Ru Group 2021)	50M
		2) Send an email		
			myMail (Mycom BV 2021)	10M
			AnyMail (Craigpark Limited 2021)	10M
	Tip Calculator	Add a bill with information of the tip	TipCalculator (Apps By Vir 2021)	
		then calculate the share of tip		
		per person		
			TipCalc (Apps By Vir 2021)	500
			Simple Tip (TLe Apps 2021)	1K
			TipCalc.Plus (ZaidiSoft 2021)	500
			FreeTipCalc. (JPStudiosonline 2021)	1K

$^{a}$ Number of downloads

### Semantic Matching in Isolation: Experimental Setting

We evaluated semantic matching in isolation (RQ1, RQ2, RQ3), by systematically evaluating all possible configurations against every individual query produced with any of the 95 subjects.

#### Configurations

**Fig. 5:**
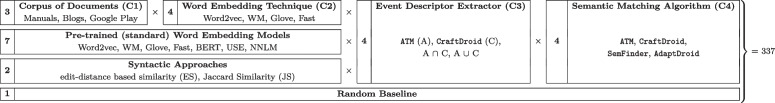
The 337 configurations of components’ instances considered in our study

Figure 5 shows the 337 configurations that we considered for answering RQ1, RQ2 and RQ3. We built the set of configurations as all the feasible combinations of the Semantic Matcher instances that we discussed in Section 3. The figure illustrates how we combined the instance to obtain 337 configurations. For example, $\langle C1= \text {\texttt {Manuals}}, C2= \text {\texttt {Word2vec}} , C3= Intersection, C4=\text {\texttt {SemFinder}} \rangle$ is a configuration out of the 337 semantic matching configurations. We experimented with 19 Word Embedding models (components C1, C2), 12 of which obtained by training four Word Embedding techniques, with three corpora of documents, seven pre-trained models, and two syntactic approaches. We combined the 19 embedding models and the two syntactic approaches with four Event Descriptor Extractor (component C3) and four Semantic Matching Algorithms (component C4). We also experimented with the random baseline.

We cleaned the corpora of documents with a standard preprocessing step (Line 2 of Algorithm 1). We considered seven Word Embedding techniques (Word2vec, WM, GloVe, FastText, *BERT, USE, NNLM*) pre-trained with the models that are provided by the authors of such techniques, and that are obtained with not-publicly-available corpora of documents (such as, different versions of Google News and Twitter datasets). We built models by training four (Word2vec, WM, GloVe and FastText) of the seven models with three corpora of documents (Manuals, Blogs, and gp). We did not build models with BERT, USE and NNLM, because these techniques require a non-trivial parameter tuning that goes beyond the scope of this paper.

We considered two canonical syntactic approaches that compute the syntactic similarity of words/sentences: Edit Distance Similarity, and Jaccard Similarity. Edit Distance Similarity computes the distance of two words $wd_1$ and $wd_2$ as

$$\begin{aligned} ES(wd_1,wd_2) = \frac{\max (|wd_1|, |wd_2|) - LD(wd_1,wd_2)}{\max (|wd_1|, |wd_2|)} \in [0 ; 1] \end{aligned}$$

where LD($wd_1$ $wd_2$) is the “Levenshtein distance” (Levenshtein 1966) of $wd_1$ and $wd_2$, that is, the minimum number of operations (deletion, insertion and substitution) required to transform $wd_1$ into $wd_2$ and vice versa. Edit Distance Similarity returns 1 if the words are identical. Edit Distance Similarity operates at word level, and thus replaces the query of the Word Embedding model at line 8 of Algorithm 1.

Jaccard Similarity computes the similarity of two sentences $\text {txt}_1$ and $\text {txt}_2$ as the number of elements that belong to both strings over the number of elements that occur in either or both strings:

$$\begin{aligned} \text {JS}(\text {txt}_1,\text {txt}_2) = \frac{|\text {txt}_1 \cap \text {txt}_2|}{|\text {txt}_1 \cup \text {txt}_2|} \in [0 ; 1] \end{aligned}$$

Jaccard Similarity returns 1 when $\text {txt}_1$ and $\text {txt}_2$ have all identical words, regardless of their position in the sentences. Jaccard Similarity operates at sentence level, and thus replaces the interrogation of the Word Embedding model at line 12 of Algorithm 1.

We experimented with the four sets of Event Descriptor Extractors summarized in Table 2 combined with the four Semantic Matching Algorithms instances. We denote descriptors and algorithms with the suffixes *“_d”* and *_a*, respectively. For instance, ATM denotes the descriptor set and ATM the algorithm of ATM, respectively. We modified the Semantic Matching Algorithms to work with sets of descriptors different from the ones used in the original algorithms, by either pruning the attributes that do not belong to the set from the algorithm or appending the new attributes at the end of the *text* attribute in the algorithm. For instance, we combine the “intersection” set with CraftDroid, by removing *activity-name*, *parent-text* and *sibling-text* from the set of attribute types at Line 32 of Algorithm 2; We combine the CraftDroid set with ATM, by appending *activity-name*, *parent-text* and *sibling-text* to the attribute *text* at Lines 16 and 20 of Algorithm 2. By appending the attributes at the end of the *text* attribute, we comply with both ATM and CraftDroid: ATM prioritizes attributes by position, with highest priority to *text*, and CraftDroid handles *text* jointly with the *hint* attribute (line 32 of Algorithm 2).

The random baseline assigns a random score between 0 and 1 to each pair of events. We repeated the experiments 100 times, to cope with the stochastic nature of the random baseline, and report the median.

#### Experimental Setup

We experimented with 95 unique scenarios, defined as pairs of source target test cases $\langle t^s, t^t\rangle$. Each scenario is paired with the ground truth that is defined as the events $e^t_{gt}$ $\in t^t$ that match the events $e^s \in t^s$. We got the ground truth for CraftDroid scenarios from the original CraftDroid paper, and we manually defined the ground truth for the ATM scenarios. The target test case $t^t$ may include ancillary events (Zhao et al. 2020), that is, events in $t^t$ that do not correspond to any event in $t^s$, and that are required to reach relevant states in the app. Since this set of research questions deal with semantic matching in isolation, we do not consider ancillary events for these questions. Some events occur multiple times in test cases for the same app. We prune redundant events, that is events that share all nine event descriptors with other events, and we obtained 337 unique queries for evaluating semantic matching.

We define the set of candidate target events $E^t =\{ e^t_0, e^t_1, \cdots e^t_n \}$ for each $e^s \in t^s$ as the set of events that are actionable in all the GUI states that the target test $t^t$ visits. More formally, $E^t = \{e^t:\exists ~S \in \mathbb {S}, e^t~\text {is actionable in }S\}$, where $\mathbb {S}$ is the sequence of state transitions obtained by executing $t^t$. Some events $t^t$ may occur multiple times in the same window and thus in $E^t$. We prune redundant events, that is events that share all nine descriptors with other events, from $E^t$. The cardinality of $E^t$ ranges from 5 to 80, with an average of 24.03 and median of 19 events. Our definition of $E^t$ leads to semantic matching queries that are coherent with Test Reuse, which matches events across applications by considering also target events that span multiple windows (Behrang and Orso 2019; Lin et al. 2019).

#### Evaluation Metrics

Our experiments are queries that score events in the target app according to their similarity with respect to events in the source test case. A query *q* sorts a set of input events $E^t$ of the target app, according their similarity score with respect to an event $e^s$ in the source test case, and returns a sorted list: $\langle e^s, E^t \rangle \xrightarrow []{q} (E^t_{sorted})$. We rank each query $q_i$ according to the position of the correct event $e^t_{gt}$ that is the event that the query should return according to the ground truth. The $rank_i$ of a query $q_i$ is the position of $e^t_{gt}$ in the list sorted according to the similarity score of $q_i$. We rank events with the same score as the average of their positions in the list.

We experimented with 337 queries, and we measured the effectiveness of the Semantic Matching Algorithms with two metrics that we compute on the returned ranks: MRR, the Mean Reciprocal Rank (Liu 2023), and Top1, the ratio of queries in which the rank of the correct answer is one.

The reciprocal rank of a query response is the multiplicative inverse of the rank of the first correct answer: 1 for first place, 1/2 for second place, 1/3 for third place and so on. The mean reciprocal rank is the average of the reciprocal ranks of our 337 queries *Q*.

$$\begin{aligned} {\textbf {\texttt {MRR}}} = \frac{1}{|Q|} \sum _{i=1}^{|Q|} \frac{1}{\text {rank}_i} \in (0 ; 1] \end{aligned}$$

MRR is a standard statistical measure for evaluating any process that produces a list of possible responses to a query *q*, sorted by their probability of correctness. MRR is suitable in our context because it focuses on a single correct answer ($e^t_{gt}$), while other metrics like Mean Average Precision (MAP) and Normalized Discounted Cumulative Gain (NDCG) focus on multiple correct answers (Liu 2023).

Top1 is the ratio of queries in which the ground truth ($e^t_{gt}$) is in the first position of the returned list. Top1 is less informative than MRR, because it ignores the position of $e^t_{gt}$ when not top in the list. However, Top1 is significative in our context, since most Test Reuse approaches choose the first event in the list.

$$\begin{aligned} {\textbf {{\texttt {Top1}}}} = \frac{1}{|Q|} \sum _{i=1}^{|Q|} \left\{ \begin{array}{lr} 1 & \text {if } \text {rank}_i = 1 \\ 0 & \text {otherwise} \end{array}\right\} \in [0 ; 1] \end{aligned}$$

#### Semantic Matching Evaluator Prototype

We evaluated the queries with the Semantic Matching Evaluator, a prototype framework that we developed in Python to evaluate semantic matching queries. Our Semantic Matching Evaluator runs different configurations of the four component types on a set of source and target events. Our Semantic Matching Evaluator is a general framework that can be configured with different choices of component types. For our experiments, we instantiated the Semantic Matching Evaluator for the configurations in Fig. 5.

The source code of ATM and CraftDroid is publicly available, ATM is written in Java, CraftDroid in Python (Lin et al. 2019). We implemented the Semantic Matching Algorithm of ATM in Python, by referring to the original Java implementation. We implemented the Semantic Matching Algorithm of CraftDroid by reusing the original Python code as much as possible (Lin et al. 2019). The Semantic Matching Algorithms of ATM and CraftDroid are internal algorithms of the Test Reuse components and can be hardly executed in isolation.

Our implementations of the Event Descriptor Extractor instances execute the source and target test cases. They retrieve the GUI state at runtime with the framework Appium (1.1.13), and extract the values of the nine widget attributes in Table 2. We implemented our own extractors, rather than rely on the implementations of ATM or CraftDroid, to have a common tool to collect all the descriptors. Our event extractor retrieves all types of click and fill events that ATM and CraftDroid use in their experiments The click events include simple click, swipe and long click, and are applicable to a wide range of Android widget types such as Button, ListView, Dialog, and ImageButton. Fill events insert a text into an EditText widget.

### Semantic Matching in Isolation: Experimental Results

We ran our 337 queries for each of the 337 configurations, for a total of 113,569 semantic matching queries. MRR ranges from 0.201 to 0.795 across all configurations with average 0.685 and quartiles Q1: 0.649, Q2: 0.693, Q3: 0.724. The original configuration of ATM [manuals (C1), w2v (C2), ATM (C3), ATM (C4)] is the 190th configuration in the list of the 337 configurations sorted according to the MRR values (MRR = 0.677). The original configuration of CraftDroid [standard (C1), w2v (C2), CraftDroid (C3), CraftDroid (C4)] is the 206th configuration in the list (MRR = 0.670).

Top1 ranges from 0.065 to 0.671 with average 0.518, and quartiles Q1: 0.465, Q2: 0.510, Q3: 0.508. The original configuration of ATM is the 244th configuration, (Top1 = 0.472), while the original configuration of CraftDroid is the 231th configuration (Top1 = 0.484).

The best configuration in both lists is [Google-Play (C1), WM (C2), ATM (C3), SemFinder (C4)] and the worst is random.

**Fig. 6:**
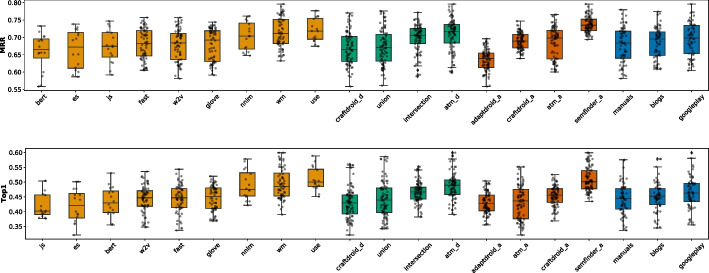
Distribution of MRR (top) and Top1 (bottom) for each component

Figure 6 shows the distributions of MRR and Top1 by instance. For example, the box plot of SemFinder on the top right of Fig. 6 shows the distribution of the MRR values of all the 84 configurations with SemFinder as the semantic matching algorithm. The box plots of the same component type are sorted by median.

The instances of the component types are unevenly distributed among the configurations. For instance, WM is present in 64 configurations, while USE only in 16. This is because for WM we considered the pre-computed standard model and three models built from the three corpora of documents, while for USE we only considered the pre-computed model.

**Table 6:** Distributions of the 337 combinations sorted by MRR and Top1

Type	Instance	MRR			Top1		
		[1:3]	[1:16]	[1:33]	[1:3]	[1:16]	[1:33]
	blogs	0%	19%	12%	0%	**15%**	12%
C1	manuals	**33%**	19%	12%	0%	**15%**	12%
	googleplay	**33%**	**31%**	**27%**	**33%**	**15%**	**19%**
	w2v	0%	0%	6%	0%	0%	0%
	glove	0%	0%	0%	0%	0%	0%
	wm	**100%**	**94%**	**56%**	**100%**	**69%**	**62%**
	fast	0%	0%	6%	0%	0%	4%
C2	bert	0%	0%	0%	0%	0%	0%
	nnlm	0%	0%	12%	0%	15%	15%
	use	0%	6%	15%	0%	15%	19%
	js	0%	0%	3%	0%	0%	0%
	es	0%	0%	0%	0%	0%	0%
	ATM_d	**100%**	**50%**	**36%**	**67%**	**46%**	**42%**
C3	CraftDroid_d	0%	19%	19%	0%	8%	19%
	intersection	0%	6%	18%	0%	0%	15%
	union	0%	25%	27%	33%	**46%**	23%
	ATM_d	0%	19%	15%	0%	6%	15%
	AdaptDroid_d	0%	0%	0%	0%	0%	0%
C4	CraftDroid_d	0%	0%	0%	0%	0%	0%
	SemFinder	**100%**	**81%**	**85%**	**100%**	**93%**	**81%**

*LEGEND::* The columns indicate the percentage of queries that locate the correct answer in positions [1:3] (1% percentile), [1:16] (5% percentile), [1:33] (10% percentile) of the list of 337 configurations sorted by MRR or Top1

Table 6 shows the distributions of the various component instances as indicated in Fig. 5 for three percentiles 1% (top 3 entries [1:3]), 5% (top 16 entries [1:16]), and 10% (top 33 entries [1:33]). The random configuration does not include any instance of components, thus it does not occur in the table. The values in the cells indicate the percentage of top 3, 16, 33 configurations that use a given instance of the components that are indicated in the rows for each group of components (C1, C2, C3, C4). We compare instances by component, thus values of each group (C1, C2, C3, C4) are mutually independent. For instance, the top three cells of the first column of Table 6 indicates that 33% of the configurations that MRR ranks in the top three use manual (C1.Manuals), and 33% use Google-Play (C1.Google-Play) for Corpus of Documents (C1 type). The cumulative percentage of each column for group type C1 is less than 100% since some top configurations use pre-trained Word Embedding models that is not in C1. The table clearly indicates the dominant role of some instances: WM, ATM and SemFinder are used by all top three configurations (100%) for C2, C3, and C4, respectively, according to MRR. Top1 confirms the full dominant role of WM and SemFinder (100%) and partially of ATM as well (67%).

We tested the pairs of instances in Table 6 for *statistical significance* using Mann-Whitney U test (Mann and Whitney 1947). We rejected the null hypothesis that the two distributions are the same with *p-value* $<= 0.05$. The null hypothesis invalidated 23 out of 51 pairs of instances from MRR metrics, and 18 for Top1 metrics. Appendix 1 reports the results of the Mann-Whitney U test.

#### RQ1: Baseline Comparison

Both MRR and Top1 ranking indicate that all configurations perform better than the random baseline. Both metrics rank random last in the sorted list, with MRR and Top1 values of 0.201 and 0.065, respectively, much lower than the configurations with the second lowest values, 0.595 and 0.359, respectively.

Table   6   indicates that syntactic based similarity metrics (Edit Distance Simila-rity and Jaccard Similarity) perform worse than Word Embedding models. Indeed, none of the 32 configurations with either Jaccard Similarity or Edit Distance Similarity appear in the top 10% configurations sorted by either MRR or Top1 values. The distribution of MRR and Top1 of Fig. 6 confirms Edit Distance Similarity and Jaccard Similarity in the leftmost side of the distribution sorted in increasing order. 
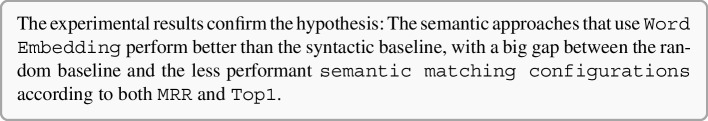


#### RQ2: Component Effectiveness in Isolation

The results reported in Table 6 and Fig. 6 indicate that the most effective instances of the four components evaluated in isolation are: Google-Play, WM, ATM, and SemFinder for each of the four components of Fig. 5. Below we discuss the evidence from experimental data in details.

**Corpus of Documents (Component C1)** Table 6 indicates that Google-Play is the Corpus of Documents that occurs more often in the top ranked combinations, according to both MRR and Top1 for all percentiles. The distribution in Fig. 6 confirms the result with the distribution of Google-Play in the rightmost position. The statistical test shows a significant difference between the approaches that use Google-Play and Manuals (MRR p-value of 0.0479), and a less significant difference between the approaches that use Google-Play and Blogs (MRR p-value of 0.0954).

We studied the impact of the Out Of Vocabulary (OOV) issue that occurs when the query involves words that do not belong to the considered corpus. We collected the OOV issues for the 36 configurations with Word2vec as Word Embedding technique, and Google-Play, Manuals, and Blogs as corpora of documents, and compared the cumulative number of OOV for the three clusters that use googleplay, manuals, and blogs, respectively. The cumulative 25,119, 364,049 and 208,075 OOV for googleplay, manuals, and blogs indicate that googleplay suffers significantly less than manuals and blog from OOV.

**Word Embedding (Component C2)** Figure 6 indicates that sentence level Word Embedding techniques, WM and USE, are the best techniques according to both MRR and Top1. The difference between sentence level (WM and USE) and word level techniques (FastText, Word2vec, GloVe, ES) is statistically significant for both MRR and Top1 (The MRR p-values of WM and word level techniques are 0.000, 0.000, 0.000, and 0.003 respectively, while the MRR p-values of USE and word level techniques are 0.001, 0.001, 0.002, and 0.007). Table 6 indicates that WM dominates USE and all other techniques. The inspection of GUI textual attributes indicates that many of them are expressed with multiple words, and this explains the better performance of sentence level over word level techniques.

Event Descriptor Extractor ** (Component C3)** Figure 6 indicates that ATM and *intersection* perform better than *union* and CraftDroid, as event descriptor selectors, and the difference is statistically significant (ATM and *intersection* differs from *union* and CraftDroid with MRR p-values of 0.000).

Table 6 confirms the dominance of ATM also in terms of occurrences in top ranked positions according to both MRR and Top1 for all percentiles.

A deep analysis of the results reveals an unbalanced distribution of the attribute types in our subjects: 8,099 source and target events define the *activity-name* attribute, 7,837 the *id* attribute, 4,532 the *text* attribute, 957 the *neighbor-text* attribute, 837 the *content-desc* attribute, 600 the *parent-text* attribute, 554 the *file-name* attribute, 165 the *sibiling-text* attribute, and no events defines the *hint* attribute. The poor performance of *union* and CraftDroid may depend on the high frequency of the *activity-name* attribute, which is defined for each event. Unrelated events in the target app that share the activity name of the source event may yield a similarity score higher than the correct match ($e^t_{gt}$), and this impact on the final score.

**Semantic Matching Algorithm (Component C4)** Figure 6 indicates that SemFinder outperforms ATM, CraftDroid, and AdaptDroid, always with statistical significance (SemFinder differs from other Semantic Matching Algorithms with MRR p-values of 0.000). Indeed, the MRR and Top1 medians of SemFinder are higher than the median of other algorithms. Table 6 confirms that SemFinder is the semantic matching algorithm that occurs more often in the top ranked combinations, according to both MRR and Top1 for all percentiles.

The analysis of the performance of the algorithms indicates AdaptDroid as the best performing approach: the configurations with AdaptDroid complete all 337 queries in 170 seconds in average, the configurations with SemFinder in 255 seconds, the configurations with CraftDroid in 393 seconds, and the configurations with ATM in 600 seconds. This suggests that combining attribute values into a single sentence can reduce the runtime while improving the results of semantic matching, as long as there are no prioritization of attributes.

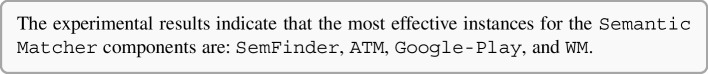



#### RQ3: Component Impact Analysis in Isolation

We studied the impact of the component types with a “local” sensitivity analysis (Crick and Hill 1987) that varies the instance of a component type at a time while holding the others fixed (Hamby 1995). We clustered the 337 configurations of the four component types, by varying an instance of a component while fixing instances of the other three components. For example, if we consider Component C2 and exclude the random baseline, we have nine possible instances. Every time we fix the values for components Component C1, Component C3, Component C4, we define a new cluster with nine configurations (in which only Component C2 varies). We compute the *standard deviation* (SD) of the MRR values of these nine configurations. This SD value represents the impact of Component C2 in the cluster (if the choice of Component C2 has high impact, the SD value is high, otherwise it is low) (Hamby 1995). SD is a measure of the amount of variation or dispersion of a set of values. A low SD indicates that the values tend to be close to the mean of the set, while a high SD indicates that the values are spread out over a wider range. This means that if a component has a high impact on the semantic matching, the SDs values of each cluster must be high. We repeated this process for component C2 48 times, that is, for every possible combination of the values of components C1, C3, and C4, obtaining $3\times 4\times 4=48$ SDs that globally capture the impact of component C2 on the semantic matching. We ran this analysis for all four component types.

**Fig. 7:**
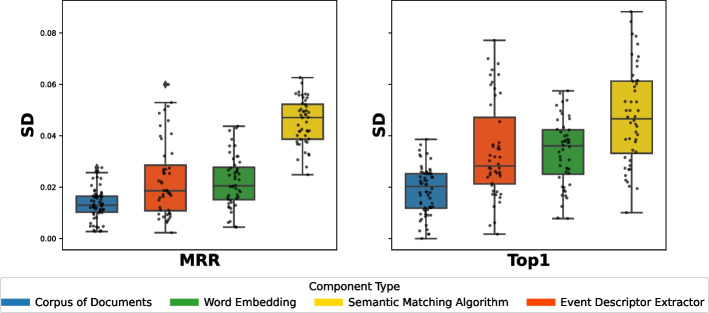
Impact analysis of components

We computed the SDs for both the MRR and the Top1 values. Figure 7 shows the distributions of the SDs values for category type, and sorts the components left to right by impact.

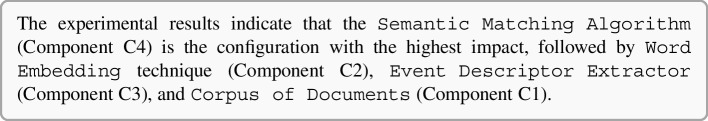



### Semantic Matching in the Context of Test Reuse: Experimental Setting

We evaluated semantic matching in the context of Test Reuse (RQ4, RQ5, RQ6) by systematically running ATM and CraftDroid with selected configurations against 89 migrations of test cases.

#### Configurations

Migrating GUI test cases is time consuming due to the cost of executing GUI events on actual apps. It takes 15 minutes on average to migrate an ATM test case on our server, and 30 minutes to migrate a CraftDroid test case, with an overall cost of over 1,000 days of computation time to investigate the 337 configurations of our experimental setup. We designed a feasible evaluation context, by sampling the semantic configurations according to a process that considers enough configurations to study every possible instance of every component: (i) We ordered the configurations based on the MRR values that we computed when studying the components in isolation, and (ii) uniformly sampled the configuration every X positions from the top. We choose X=5 that is the highest sampling step that guarantees all the semantic matching instances to be included at least once in the study. This process selected 68 configurations. We also considered random and perfect configurations. The random configuration assigns a random score between 0 and 1 to each pair of events, and serves as the baseline, to quantify the impact of semantic based approaches. The perfect configuration assigns score 1 to the correct pairs of events, based on the ground truth, 0 otherwise, and represent the target for test reuse approaches.

ATM considers events with a similarity score greater than a threshold as matching events (line 91 in Algorithm 6). The original ATM paper (Behrang and Orso 2019) uses a threshold optimized for the configuration considered in the paper. Our experiments indicate that the same threshold may penalize the results of ATM for other configurations and applications. Thus we decided to use different thresholds across configurations and applications.

We derived unbiased thresholds for each pair of applications and each configuration from the similarity scores computed with respect to the considered configuration for the pairs of events that occur in the other applications. Intuitively, we compute the threshold for each pair of applications $\langle A^i, A^j \rangle$ as the similarity score that best separates correct from incorrect pairs of events, by considering all pairs of applications that do not include either $A^s$ or $A^t$. In details, we consider the similarity scores computed for all pairs of events in the experiment in-isolation. We computed the threshold of each pair of applications $\langle A^i, A^j \rangle$ from the similarity score of all pairs of events that occur in any pair of applications, but the pairs that include either $A^s$ or $A^t$. We compute the threshold as the similarity score that best separates the pairs of correct and incorrect matches, that is, the score that maximizes the F1-score computed for pairs above and below the threshold.

We computed the threshold for all configurations that differ in the Corpus of Documents, Word Embedding and Semantic Matching Algorithm, components. We did not distinguish configurations that differ in Event Descriptor Extractor and Event Selector, since the semantic score does not depend on Event Selector, and only partially on Event Descriptor Extractor.

#### Experimental Setup

We experimented with 34 unique scenarios, 11 ATM and 23 CraftDroid scenarios. Each scenario is a test case $t^s$ of the source application with the ground truth $t^{gt}$, and a target application. The ground truth indicates the expected matching, as provided by the CraftDroid authors for CraftDroid test cases, and manually identified by us for the ATM test cases.

**Fig. 8:**
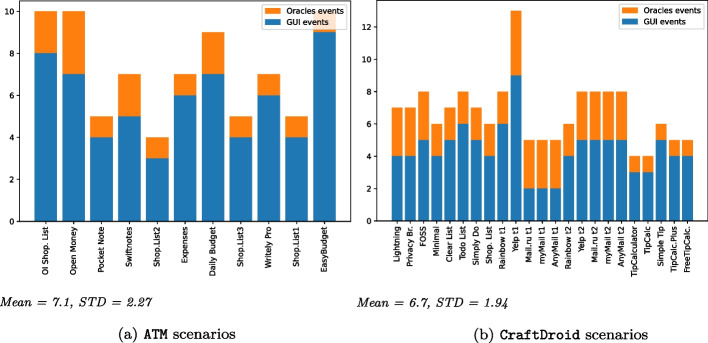
Size of the source test cases

**Fig. 9:**
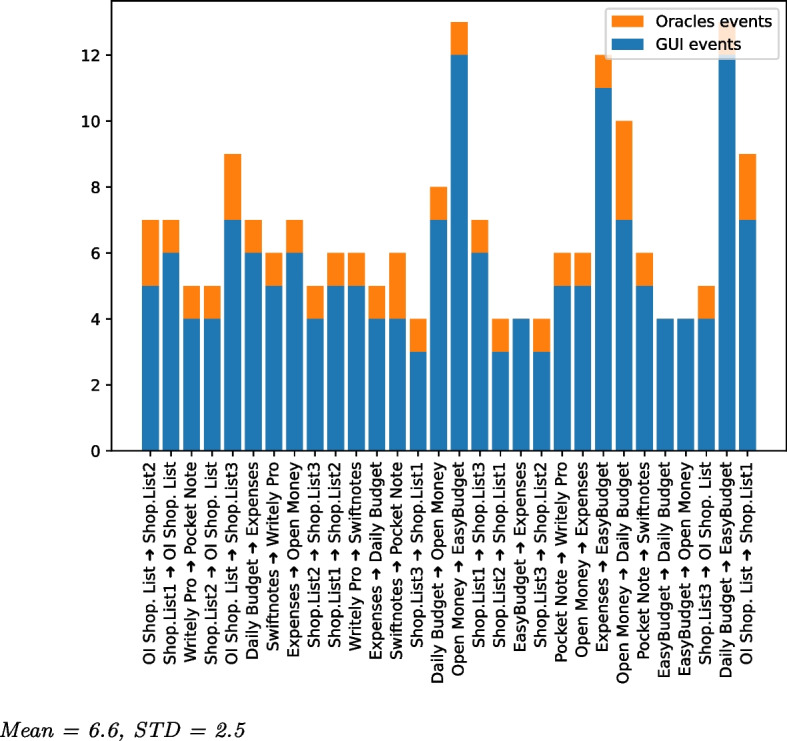
Size of the ground truth for ATM scenarios

Figure 8(a) and (b) show the size of the ATM and CraftDroid test cases in terms of number of GUI events. Figure 9 shows the size of the ground truth of ATM scenarios, that is, the number of events that belong to the correct mappings across test cases of compatible applications. CraftDroid refers to the source test cases as the ground truth of compatible applications, thus the size of the ground truth is equal to the size of the source test cases. The figures explicitly distinguish events that correspond to assertions (oracle events). We consider both GUI and oracle events, since Test Reuse approaches migrate both GUI and oracle events with semantic matching. Oracle events commonly occur at the end of the test cases, thus the possibility of generating oracles depends on the ability to migrate all previous events in the test case. We evaluate the migrated test cases with oracles included and oracles excluded for an unbiased evaluation of both GUI and oracle events.

We assessed semantic matching in the context of Test Reuse, by comparing the quality of the test cases that ATM and CraftDroid migrate with the 68 sampled configurations. We executed some sample configurations for five hours, and we identified an upper bound for each run as the maximum execution time after which no tool migrates test cases. We set the maximum execution time to 2.5 hours.

#### Evaluation Metrics

We evaluate the quality of the migrated test cases as the fidelity of two associations: the *source-to-ground-truth* and the *ground-truth-to-migrated* associations. The *source-to-ground-truth* association maps source test cases to ground truth test cases. It indicates the sequence of events in the source test case for the source app that shall be migrated to obtain an optimal test for the target app. The *ground-truth-to-generated* association maps ground truth test cases to their corresponding migrated test cases. It indicates the events of the source app that are correctly mapped to the target app.

The *source-to-ground-truth* association is defined manually once for all by the authors of the considered subject cases (Section 4.1). Given a source test case $t^s = \langle e^s_0, e^s_1, \cdots e^s_k\rangle$ and a ground truth test case $t^{gt} = \langle e^{gt}_0, e^{gt}_1, \cdots e^{gt}_m\rangle$, we refer to this association as $stg: t^s \rightarrow t^{gt}$^3^, such that, $stg(e^{s}_i)=e^{gt}_j$.

Our Fidelity plug-in builds the *ground-truth-to-generated* association $$m:t^{gt} \rightarrow t^t$$ automatically as follow: Each event in the target test case is associated with the event in the target test case that shares the values of the identifier attributes, following their order of occurrence in the grounds truth test case. More rigorously, Let $$t^{gt} = \langle e^{gt}_0, e^{gt}_1, \cdots e^{gt}_m\rangle $$ and $$t^t = \langle e^t_0, e^t_1, \cdots e^t_n\rangle $$ be test cases and $$a( e^j_i)$$ be the attributes of event $$ e^j_i$$, the *ground-truth-to-generated* association is a partial function $$m:t^{gt} \rightarrow t^t$$ that associates events in the ground truth test case to events in the target test case according to the following rule:

$$m(e^{tg}_i)=e^t_j$$ iff $$a(e^{tg}_i)=a(e^t_j) \wedge \forall w<j, \exists k<i \ | \ a(e^{tg}_i) \ne a(e^t_w) \vee m(e^{tg}_k) = e^t_w$$

Let us define, $m(t^{gt})=\bigcup _i m(e^{gt}_i)$, that is, the set of events in the target test case that are associated with any event in the ground truth test case.

We measure the fidelity of the associations with the F1-Score fidelity metric that we compute from *Precision, Recall* as in FrUITeR (Zhao et al. 2020):

$$\begin{aligned} Precision = \frac{TP}{TP + FP} \end{aligned}$$

$$\begin{aligned} Recall = \frac{TP}{TP + FN} \end{aligned}$$

$$\text {F1-score } = \frac{2 \times Recall \times Precision}{Recall + Precision} $$

where we define true positives (TP), false positives (FP), and false negatives (FN) for a source test case $t^s$, a target test case $t^t$, and a ground truth $t^{gt}$, and the related *stg* and *m* associations, as follows:

TP: the cardinality of *ground-truth-to-migrated*, that is, the number of events in the source test case that are correctly mapped according to the ground truth:
  $$\begin{aligned} TP=|{m(t^{gt})}| \end{aligned}$$
FP: the cardinality of the difference between the migrated test and the *ground-truth-to-migrated* association, that is, the number of events in the migrated test case that do not exist in the ground truth:
  $$\begin{aligned} FP=|{t^t \setminus m(t^{gt})}| \end{aligned}$$
FN: the cardinality of the difference between the *source-to-ground-truth* and *ground-truth-to-migrated* associations, that is, the number of events in the source test case that have an equivalent event in the ground truth, and are missed in the migrated test case.
  $$\begin{aligned} FN=|{stg(t^s)\setminus m(t^{gt})}| \end{aligned}$$

FrUITeR computes the fidelity of the mappings between the source and migrated test cases by instrumenting the Test Reuse approaches. We compute the fidelity between source and ground-truth test cases and between ground-truth and migrated test case to avoid the instrumentation overhead.

#### Prototype Test Migration Evaluator

We evaluated the test migration with The Test Migration Evaluator, a prototype framework that integrates the Semantic Matching Evaluator with both the ATM and CraftDroid Test Generator, as well as with a Fidelity plug-in that measures the fidelity of the source and migrated test cases with respect to the ground truth.

We implemented our plug-in in Python, instead of extending FrUITeR (Zhao et al. 2020), because (i) FrUITeR requires transforming the test cases into a canonical format, and the transformers are available for Java test cases only, while CraftDroid test cases are in *JSON* format, and (ii) FrUITeR identifies events by *resource-id* or *XPath* that do not uniquely identify events across the migration process: *resource-id* can be shared across multiple widgets, and the migration process generates different *XPath* for the same events.

TheTest Migration Evaluator gets a set of pairs of source and target applications, with a set of test cases for each of the source application, and any subsets of the 337 configurations considered in our study. It migrates the input test cases according to input configurations.

We integrated ATM and CraftDroid in our framework, by interfacing both the ATM and CraftDroid  Event Selector with our Semantic Matching Evaluator. In this way, we use the same Semantic Matcher with both ATM and CraftDroid. OurTest Migration Evaluator pairs both ATM and CraftDroid with all the Event Descriptor Extractor strategies implemented in the Semantic Mat-ching Evaluator. For instance, our Test Migration Evaluator can evaluate the CraftDroid generation approach with file-names attribute, thus extending the original CraftDroid approach.

**Table 7:** Correlation of semantic matching metrics and test reuse metric, with oracles

	CraftDroid		ATM		CraftDroid	
Subjects	All		Shared		Shared	
Metric	F1 MRR	F1 Top1	F1 MRR	F1 Top1	F1 MRR	F1 Top1
Correlation	0.39063 (M)	0.51028 (S)	0.33981 (M)	0.42692 (M)	0.1505 (-)	0.22328 (-)
p-value	0.00099	0.00001	0.00458	0.00028	0.22057	0.06721

*S = strong correlation, M = moderate correlation, W = weak correlation, - = statistically insignificant*

### Semantic Matching in the context of test reuse: Experimental Results

We migrated all 89 scenarios, that is, test cases with the ground truth, and target application, for the 68 selected configurations, for a total of over 6,000 migrations.

#### RQ4. Impact of Semantic Matching in the Context of Test Reuse

We measure the impact of semantic matching on test reuse as the correlation between semantic matching metrics (MRR and Top1) and test reuse metrics (F1-Score). The experimental results indicate a statistically significant impact of semantic matching on test reuse, and similar performance of most configurations on both ATM and CraftDroid test reuse approaches.

Tables 7 and 8 show the Pearson correlation (Pearson 1931) between the MRR and Top1 metric values for semantic matching, on one side, and the F1-Score for test reuse, on the other side. The result includes 68 values for F1-Score and 68 for MRR. The full results are available in the replication package. The tables report the correlations from experimenting with CraftDroid on both *all scenarios* and *shared scenarios*. They report the correlations from experimenting with ATM on the shared scenarios only, since we cannot adapt ATM for all subjects. The tables report also the p-values that we compute to validate the statistical significance of the results. We indicate the correlation as weak ($\le$0.3), moderate (0.3 - 0.5), or strong ($\ge$0.5), following the widely accepted classification of Cohen (Cohen 2013), and we indicate as statistically insignificant (-) results with p-vales smaller than 0.05. The results indicate an either medium or strong correlation for all statistically significant cases. The results in the tables indicate that oracles do not impact significantly on the correlation.

**Fig. 10:**
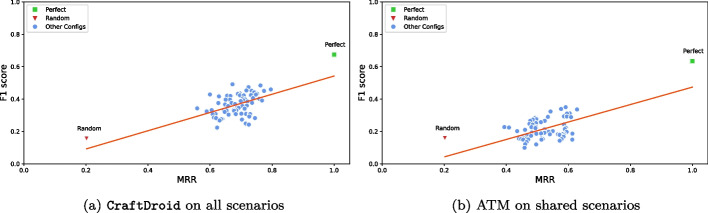
Correlation between semantic matching (MRR) and Test Reuse (F1-Score) with oracles

**Fig. 11:**
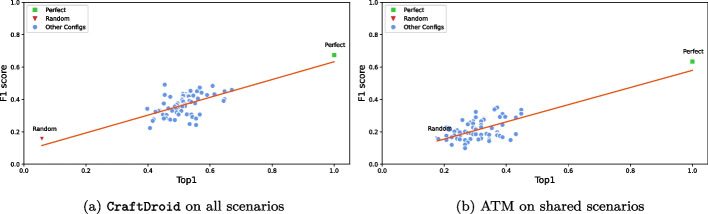
Correlation between semantic matching (Top1) and Test Reuse (F1-Score) with oracles

Figures 10 and 11 plot the MRR and Top1 metrics with respect to the F1-Score for both CraftDroid and ATM executed with all and shared scenarios, respectively. The figures report also the correlations for the perfect (green square dots) and random (red triangle dots) baselines. The F1-Score of the perfect configuration is 0.6627 for CraftDroid and 0.557 for ATM. The F1-Score of the semantic matching is not the only factor for test migration; Test Generator plays an important role as well. The F1-Score of the random configuration is 0.1553 for CraftDroid and 0.1698 for ATM, less than most semantic matching configurations (blue) dots, thus confirming the effectiveness of semantic matching for Test Reuse. The least squares polynomial fit with degree of one  (Gergonne 1974) (red lines) indicates the trends, which are positive in all cases, confirming the correlation between MRR and Top1 metrics. The gap between semantic matching configurations (blue) and perfect (green) dots suggests a space for improving semantic matching for Test Reuse. The results for Top1 are skewed to the left comparing to MRR, since Top1 scores 1 only if the correct candidate is exactly in the top position of the ranking while MRR scores positively also when the candidate occur in a high position that may be different from the top position. Thus Top1 does not consider the case of many good but not perfect candidates.

**Table 8:** Correlation of semantic matching metric and test reuse metric without oracles

	CraftDroid		ATM		CraftDroid	
Subjects	All		Shared		Shared	
Metric	F1 MRR	F1 Top1	F1 MRR	F1 Top1	F1 MRR	F1 Top1
Correlation	0.39830 (M)	0.49938 (S)	0.34433 (M)	0.45241 (M)	0.15759 (-)	0.24503 (-)
p-value	0.00077	0.00001	0.00404	0.00011	0.19934	0.04402

*S = strong correlation, M = moderate correlation, W = weak correlation, - = statestically insignificant*

We measure the effectiveness of semantic matching across ATM and CraftDroid configurations as the difference $\varDelta$ between the F1-Score values of ATM and CraftDroid migrations for all common configurations. We normalize $\varDelta$ values for each approach, separately, and use a tailed t-test to determine if a mean of $\varDelta$ is different from zero with p-value of 0.05. Non-zero mean values of $\varDelta$ indicate configurations with different performance for ATM and CraftDroid: ATM better than CraftDroid for positive $\varDelta$ and vice versa for negative $\varDelta$.

**Fig. 12:**
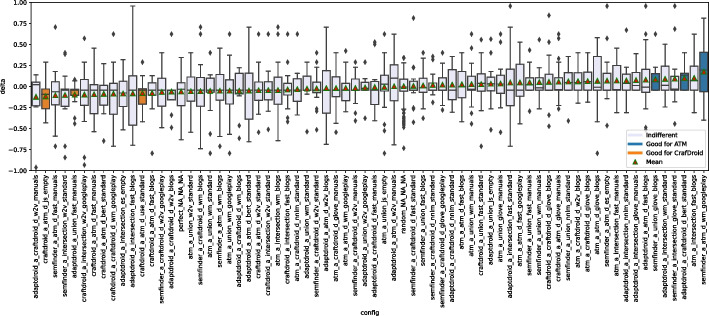
Configurations sorted by normalized $\varDelta$ of F1-Score

Figure 12 plots all configurations sorted by normalized $\varDelta$ values, and marks the negative (orange) and positive (blue) mean values. ATM and CraftDroid do not differ for most (average $\varDelta =0$) but six configurations: Three orange configurations on the left hand side (CraftDroid better than ATM), and three blue on the right hand side (ATM better than CraftDroid). Only the leftmost out of the six configurations that work better on either approaches corresponds to a p-value less than 0.001. Thus the data indicate that no specific pattern between the configurations works better for one of two approaches.

**Fig. 13:**
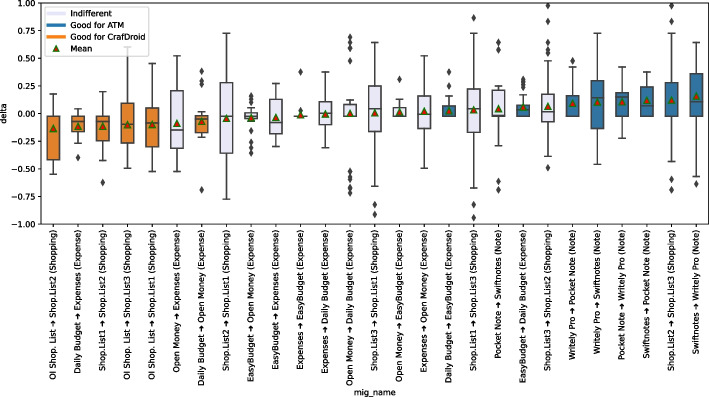
Scenarios sorted by normalized $\varDelta$ of F1-Score

We compare ATM and CraftDroid on 27 scenarios, all the scenarios that we can reproduce for both Test Generators (Shared Scenarios). Figure 13 compares the performance of the Test Generators, ATM and CraftDroid, for the different scenarios. It plots the scenarios by normalized $\varDelta$ values, to investigate the differences in performance of the Test Generators in various scenarios. We compute the $\varDelta$ values as in Fig. 12. The figure shows that CraftDroid performs better than ATM for six scenarios (orange in the figure), ATM for eight (blue in the figure), and the two Test Generators perform similarly for thirteen scenarios (light pink in the figure). CraftDroid outperforms ATM on *Shopping* (four) and *Expense* (two) scenarios. ATM outperforms CraftDroid on *Note* (five), *Expense* (two), and *Shopping* (one) scenarios. The relatively high number of scenarios where the Test Generators perform similarly, and a shared category of scenarios (*Expense*) where either of the approach perform better suggests that scenarios may have at most a mild impact on the choice of the Test Generator. 
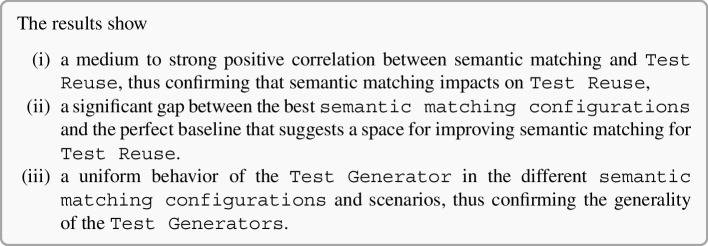


#### RQ5. Component Effectiveness in the Context of Test Reuse

We measured the effectiveness of the components (Semantic Matching Algorithm (C4), Event Descriptor Extractor (C3), Word Embedding (C2), Corpus of Documents (C1)) on Test Reuse, by ranking the scenarios grouped by component instances, according to the median F1-Score.

The experimental results indicate: (i) A more stable performance of SemFinder and AdaptDroid across subjects than ATM and CraftDroid Semantic Matching Algorithm; (ii) A clear ranking with ATM on top followed by Intersection, Union and CraftDroid as Event Descriptor Extractor, in this order; (iii) A better performance of WM and FastText over Word2vec and GloVe Word Embedding; (iv) Google-Play as the best Corpus of Documents for CraftDroid, with no relevant differences among corpora for ATM.

#### Semantic Matching Algorithm

Table 9 reports the median F1-Score for the CraftDroid and ATM configurations grouped accroding to the Semantic Matching Algorithm component for both ‘all’ and ‘shared’ scenarios. The best values are in boldface, the worst in italics. The values for ‘all’ subjects benefit from the large size of the experiments. The homogeneity of values across configurations indicates an even behavior of the different choices of the Semantic Matching Algorithm component on Test Reuse. The CraftDroid algorithm works best for the CraftDroid  Test Generator and worst for the ATM  Test Generator, while ATM works well for ATM  Test Generator and worst for CraftDroid  Test Generator. The SemFinder and AdaptDroid algorithms work stably well with both approaches on all subject. The values from the migration of test cases with and without oracles indicate a positive impact of the assertion events.

**Table 9:** Median F1-Score values of MRR grouped by Semantic Matching Algorithm

		CraftDroid			CraftDroid			ATM		
Subjects		All			Shared			Shared		
Comp.	# S.	oracles	no oracles	rank	oracles	no oracles	rank	oracles	no oracles	rank
CraftDroid	13	**0.3974**	**0.4978**	1	**0.1936**	**0.2219**	1	*0.1782*	*0.2018*	4
SemFinder	18	0.3678	0.4503	2	0.1761	0.2050	3	0.1847	0.2160	3
AdaptDroid_a	20	0.3496	0.4369	3	0.1841	0.2174	2	**0.2251**	**0.2498**	1
ATM_a	17	*0.3336*	*0.4192*	4	*0.1741*	*0.1963*	4	0.2127	0.2354	2

**Table 10:** Median F1-Score values of MRR grouped by Event Descriptor Extractor

		CraftDroid			CraftDroid			ATM		
Subjects		All			Shared			Shared		
Comp.	# S.	oracles	no oracles	rank	oracles	no oracles	rank	oracles	no oracles	rank
ATM	19	**0.4214**	**0.5169**	1	**0.2539**	**0.2888**	1	**0.2729**	**0.3036**	1
Intersection	14	0.3581	0.4434	2	0.1786	0.2072	2	0.2274	0.2680	2
Union	16	0.3316	0.4115	3	0.1656	*0.1644*	3,4	0.1888	0.2109	3
CraftDroid	19	*0.3070*	*0.3712*	4	0.1428	*0.2050*	4,3	*0.1818*	*0.2081*	4

Event Descriptor Extractor Table 10 reports the median F1-Score for the CraftDroid and ATM configurations grouped by the Event Descriptor Extractor component. The median F1-Score values in the table clearly indicate a ranking: ATM, Intersection, Union, CraftDroid, with and without oracles, and for all sets of configurations, for both ATM and CraftDroid but CraftDroid with oracles on shared subjects.

Word Embedding Table 11 reports the median F1-Score for the CraftDroid and ATM configurations grouped by the Word Embedding component. The table reports the values for the instances that occur at least ten times in the sampled configurations: WM, GloVe, FastText, and Word2vec. The values indicate that WM and FastText perform better than Word2vec and GloVe.

**Fig. 14:**
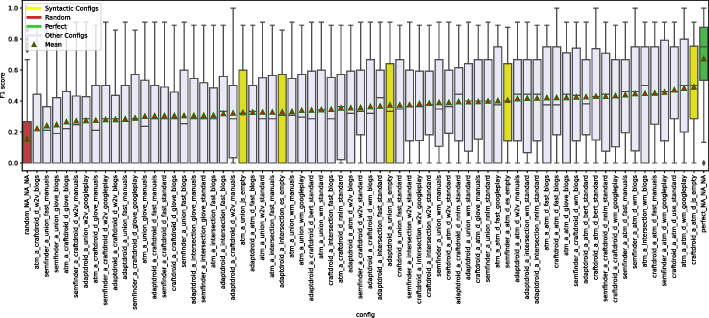
Range of F1-Score per semantic matching configurations in CraftDroid with all scenarios, Ordered by mean

**Fig. 15:**
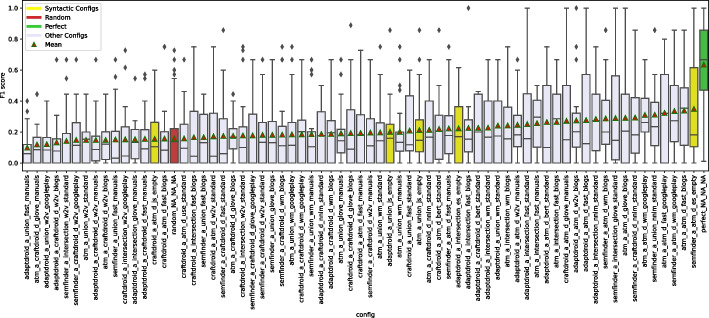
Range of F1-Score per semantic matching configurations in ATM with shared scenarios, Ordered by mean

Figures 14 and 15 compare syntactic and semantic configurations, by plotting the configurations sorted by mean of F1-Score. We aggregate semantic configurations by means since both MRR and Top1 aggregate the results by means. The yellow box plots indicate the five configurations that implement syntactic techniques, the green box plot indicates the perfect configuration, and the red box plot the random configuration. The distribution of syntactic configurations in both figures indicates no relevant differences between syntactic and semantic configurations, however the limited amount of syntactic configurations does not allow us to generalize the results. While the perfect configuration subsumes all configurations, the random configuration subsumes some configurations for ATM. This explains the lower F1-Score values for ATM than CraftDroid in Table 11.

**Table 11:** Median F1-Score values of MRR grouped by Word Embedding

		CraftDroid			CraftDroid			ATM		
Subjects		All			Shared			Shared		
Comp.	# S.	oracles	no oracles	rank	oracles	no oracles	rank	oracles	no oracles	rank
WM	13	**0.4190**	**0.5023**	1	**0.2019**	**0.2372**	1	0.2017	0.2234	2
FastText	17	0.3302	0.4192	3	0.1722	0.2153	3	**0.2099**	**0.2352**	1
W2V	14	0.3432	0.4236	2	0.1880	0.2197	2	*0.1513*	*0.1694*	4
GloVE	10	*0.3040*	*0.3700*	4	*0.1100*	*0.1224*	4	0.1870	0.2071	3

Corpus of Documents Table 12 reports the median F1-Score for the CraftDroid and ATM configurations grouped by the Corpus of Documents component. The values in the table indicate the independence of ATM from the choice of Corpus of Documents, while CraftDroid Test Generator perform best for Google-Play and worst for Manuals. 
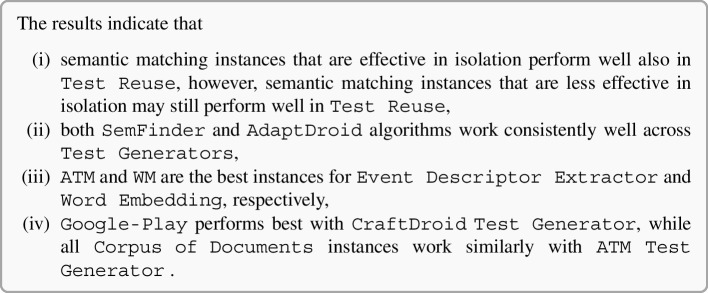

#### RQ6. Component Impact Analysis in the Context of Test Reuse

Table 13 reports the median F1-Score for both CraftDroid and ATM configurations grouped by components. The values in the table indicate that Event Descriptor Extractor is the most impactful component on Test Reuse, with F1-Score values far higher than the other components for all data sets. Semantic Matching Algorithm, and Word Embedding follow Event Descriptor Extractor with different comparative performance depending on the approach and subjects. The F1-Score values ranks Corpus of Documents as the least impactful component on Test Reuse for all setups.

**Fig. 16:**
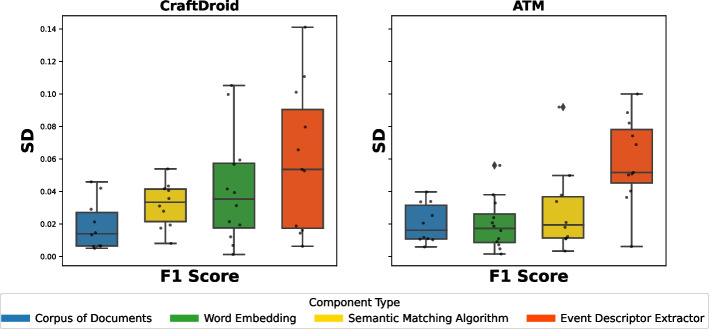
Impact analysis of component for CraftDroid Test Generator on all subject and ATM Test Generator on shared subjects

The results in the table depend on the subjects: Semantic Matching Algorithm ranks third for CraftDroid with shared subjects, and fourth with all subjects. The results with all subjects indicate a similar impact of Semantic Matching Algorithm and Word Embedding for CraftDroid. The plots in Fig. 16 confirm the similar performance of Semantic Matching Algorithm and Word Embedding for CraftDroid. 
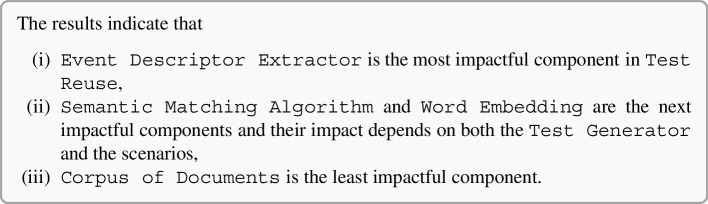

**Table 12:** Median F1-Score values of MRR grouped by Corpus of Documents

		CraftDroid			CraftDroid			ATM		
Subjects		All			Shared			Shared		
Comp.	# S.	oracles	no oracles	rank	oracles	no oracles	rank	oracles	no oracles	rank
Google Play	9	**0.3848**	**0.4772**	1	**0.2019**	**0.2296**	1	*0.1851*	*0.2084*	3
Blogs	18	0.3382	0.4223	2	0.1804	0.2111	2	0.1906	**0.2233**	2,1
Manuals	15	*0.3234*	*0.3746*	3	*0.1548*	*0.1763*	3	**0.1916**	0.2190	1,2

### Discussion

The experimental results that we discuss in this section indicate the importance of semantic matching in test reuse. They also show a substantial gap between the best semantic matching configurations and the perfect mapping (Figs. 14 and 15). The gap indicates space for improvement.

The results clearly indicate the contribution of the different instances for the semantic matching components. Here we compare the results of the experiment of semantic matching in isolation (RQ1, RQ2 and RQ3) that we discuss in detail in Section 4.3, to the results in the context of test reuse (RQ4, RQ5 and RQ6) that we discuss in detail in Section 4.5. Since different metrics captured the effectiveness of semantic matching in isolation (MRR and Top1) and in the context of test reuses (F1-Score), we compare the results qualitatively.

- Semantic Matching Algorithm **:** The evaluation in isolation indicates that SemFinder performs best and AdaptDroid worst among the evaluated Semantic Matching Algorithm. The evaluation in the context of test reuse does not reveal substantial differences among the evaluated semantic matching algorithms, all of which perform well. SemFinder and AdaptDroid semantic matching algorithms are not paired with a specific test generation approach, and perform evenly well when paired with any Test Generator. ATM and CraftDroid semantic matching algorithms perform best when paired with the corresponding test generator, worst otherwise. These results suggest that SemFinder * could be a safe choice since it performed well in all contexts*, regardless of the test generation approach used.
- Event Descriptor Extractor **:** Both the evaluation in isolation and in the context of test reuse indicate that event descriptor extractors perform well, with ATM * outperforming the others*.
- Word Embedding **:** Both the evaluation in isolation and in the context of test reuse indicate that WM performs better than the other word embeddings. FastText performs well, although often worse than, only the context of test reuse. We speculate that this result might derive from the capability of FastText to handle out-of-vocabulary issues more effectively than the other word embedding that we consider in the study, being out-of-vocabulary a phenomenon that occurs often when processing a large variety of diverse words. Overall, *the results clearly indicate* *WM* *as the best option for the task of semantic matching, among* the considered models.
- Corpus of Documents **:** Both the evaluation in isolation and in the context of test reuse suggests that *the best corpora to use may depend on how the corpora is used,* *and this on the test reuse technique*. In fact, Google-Play is the best corpora for CraftDroid, while it performs worst for ATM.
- Impact of the components on test reuse: The four components (Corpus of Documents, Word Embedding, Semantic Matching Algorithm, Event Descriptor Extractor) have a different impact on test reuse, depending on the test generator (ATM, CraftDroid), and the impact varies from evaluation in isolation and in the context of test reuse (Figs. 7, and 16). The evaluation in isolation ranks Semantic Matching Algorithm first, then Word Embedding, Event Descriptor Extractor and Corpus of Documents. The evaluation in the context of test reuse ranks Event Descriptor Extractor first, then Semantic Matching Algorithm and Word Embedding between the second and third place depending on the test reuse approach, and finally Corpus of Documents. From these results we can deduce that *the set of extracted descriptors and the way these descriptors are processed are the most important and impactful part of the semantic matching process*. While, the Word Embedding, and even more the choice of the Corpus of Documents, play a minor role in the semantic matching process. Interestingly, results also highlight how there is a gap to fill with respect to the performance of the ideal semantic matching process.

**Table 13:** Median F1-Score values of MRR grouped by Components

		CraftDroid			CraftDroid			ATM		
Subjects		All			Shared			Shared		
Comp.	# S.	oracles	no oracles	rank	oracles	no oracles	rank	oracles	no oracles	rank
Event Descriptor Extractor	11	**0.0536**	**0.0835**	1	**0.0688**	**0.0779**	1	**0.0517**	**0.0704**	1
Word Embedding	12	0.0352	0.0436	2	0.0360	0.0481	3	0.0173	0.0226	3
Semantic Matching Algorithm	10	0.0333	0.0293	3	0.0538	0.0574	2	0.0195	0.0289	2
Corpus of Documents	10	*0.0139*	*0.0245*	4	*0.0299*	*0.0331*	4	*0.0161*	*0.0189*	4

### Threats to Validity

We discuss the main threats to the validity of the results, and summarize how we mitigated them.

**External Validity** The main threat to the external validity of our results concerns the generalizability of the results to other Android apps and test migration scenarios. We mitigated this threat by experimenting with a large number of test migration scenarios (147), which is comparable to or higher than the number of scenarios that the authors of test reuse approaches used so far to evaluate their approaches (Lin et al. 2019; Behrang and Orso 2019). Moreover, we collected the subjects from two benchmark datasets built by two independent teams, spanning several app categories and functionalities (Table 5). The number of Android apps (30) that we considered in our experiments is higher than the number of Android apps used in FrUITeR’s experiments (20) (Zhao et al. 2020).

**Internal Validity** The main threats to the internal validity of our results concern both errors in our implementation that may lead to incorrect results and errors in our re-implementation of the semantic matching algorithms of ATM and CraftDroid. We mitigated the threat that may derive from errors in the implementation of Semantic Matching Evaluator (research questions RQ1, RQ2 and RQ3) by manually validating the descriptors and metrics on a set of 30 sample queries that we selected with the following characteristics: empty descriptors, and abnormally high or low MRR and Top1 values. For such queries, we manually inspect the GUI of the app to check that both the descriptors are correctly extracted and the embedding models return the computed similarity scores. We mitigated the threat that may derive from errors in the implementation of Test Migration Evaluator (research questions RQ4, RQ5, RQ6), by checking that the test generation approaches ATM and CraftDroid integrated into our framework with their original configuration, return similar results to those obtained by running the original implementations on the same subjects. We mitigated the treat that our re-implementation of the semantic matching algorithms of ATM and CraftDroid may not faithfully implement the semantic matching algorithms of ATM and CraftDroid, by referring to the original source code of the implementations of the authors of ATM and CraftDroid, to exclude that other factors besides the selection of test reuse components might affect the results. We also released our data and scripts^4^, and we welcome external validation.

**Construct Validity** The main threat to the construct validity concerns the adequacy of the measures that we use in the experiments, for evaluating the effectiveness of semantic matching and test reuse. We mitigated the threat that may derive for metrics, by considering well-established metrics. We evaluate semantic matching in isolation with MRR and Top1, widely used metrics in information retrieval. We evaluate the effectiveness of test migration with the Fidelity metrics (Zhao et al. 2020), which are the de-facto standard metrics for evaluating test reuse approaches (Zhao et al. 2020).

## Related Work

This paper presents the first study of semantic matching of GUI events for test reuse approaches. The closest related work is FrUITeR (Zhao et al. 2020), a framework that Zhao et al. designed to comparatively evaluate test reuse techniques. FrUITeR targets the end-to-end effectiveness of Test Reuse approaches by automatically evaluating the quality of the generated test cases (using fidelity metrics). Differently, our framework enables the evaluation of the semantic matching in isolation using the MRR and Top1 metrics, and also allows the automated evaluation of different combinations of the test reuse components. In particular, our study evaluates both the end-to-end effectiveness of Test Reuse techniques and the impact of each component in isolation. In our study, we identify a common workflow of test reuse approaches and propose a component-based evaluation framework that uses metrics introduced in FrUITeR. Differently from Zhao et al.’s study, we investigate the impact of each component of test reuse, and we suggest which instances of components are more effective based on the results. We conduct our semantic matching study both in isolation from and with respect to test generation, reporting novel findings and insights.

Some studies in the NLP community compare various word embedding techniques (Baroni et al. 2014; Wang et al. 2018; Li et al. 2018). Li et al. report that word embedding techniques trained on domain-specific corpora perform better on the related specialized tasks (Li et al. 2018), which is in line with the results of this paper. However, our study is the first study that compares word embedding techniques in the context of GUI events matching. In our previous work (Khalili et al. 2022) we studied the impact of domain-specific corpora on training word embedding models for the semantic matching of GUI events. The results suggest that there is an ideal level of specialization and further specializations have a negative impact.

In this paper, we propose SemFinder, a new instance of Semantic Matching Algorithm component, which addresses the limitations of current approaches. Similarly to current semantic matching algorithms (Behrang and Orso 2019; Lin et al. 2019; Mariani et al. 2021), Semantic Matching Algorithm exploits textual attributes of GUI widgets and scores target events based on their similarity to a source event. However, SemFinder addresses the main limitations of current semantic matching algorithms: (a) They consider too much information, which might include noise; (b) They restrict information and lose relevant information; (c) They miss contextual information by considering attributes separately. SemFinder balances different strategies to address these limitations.

In this paper we consider three stat-of-the-art Test Reuse approaches for Android platform (ATM Behrang and Orso 2019, 2020), CraftDroid (Lin et al. 2019) and AdaptDroid Mariani et al. 2021). We do not consider test reuse techniques that target different environments, for instance Web apps (Rau et al. 2018a, b) make different assumptions, or target different objectives. We consider the general ATM approach that migrates test cases across different apps, and not the specialized GUITestMigrator (Behrang and Orso 2018) approach that migrates test cases across apps with the same specification. We do not consider approaches to adapt GUI test cases across the Android and iOS implementations of the same app (Qin et al. 2019; Talebipour et al. 2021).

Some approaches generate Android test cases based on some predefined interaction patterns (sequence of recurrent events in abstract or concrete form). These approaches use semantic matching to find appropriate events of abstract patterns in the target application. AppFlow uses machine learning to recognize common widgets and screens (Hu et al. 2018). Augusto uses Alloy to encode patterns of prevalent functionalities among different domains (Mariani et al. 2018). These approaches use semantic matching to infer sequences of events that match some predefined interaction patterns, a problem related to but different from the use of semantic matching that we study in this paper. The findings and insights that we report in this paper can shed new light also on the use of semantic matching to find appropriate events of abstract patterns in the target application.

Some techniques automatically infer interaction patterns from sequences of GUI events (Mao et al. 2017; Zhao et al. 2022; Mao et al. 2022). Polarize (Mao et al. 2017) extracts *motifs*, patterns of recurrent concrete events, from large sets of execution traces of multiple apps, and feed the identified *motifs* to a genetic algorithm, to generate test cases for other apps, aiming to maximize coverage. Polarize uses syntactic checks to match pattern elements to the target app events. Mao et al. (2022) mine patterns that contain abstract elements, from user traces, to generate test cases for applications with similar functionalities. They use a Semantic Matching Algorithm similar to the one of CraftDroid to extract abstract element of patterns and match them to target application events. AVGUST (Zhao et al. 2022) leverages computer vision and NLP techniques to extract usage patterns from screen recordings of multiple apps. AVGUST is a developer-in-the-loop technique: It recommends top events to developers to choose from. AVGUST uses a classifier to match target events to the elements.

Qin et al. (2019) and Lin et al. (2022) use semantic matching for automatically migrating test cases for the same apps across different platforms. TestMig (Qin et al. 2019) reuses test cases of IOS apps to generate test cases for the Android version of the same apps. TestMig uses *tf-idf* to convert text to vectors of real numbers, and uses cosine similarity formula to compute the similarity of events between the two versions of the same app. Then, it maps events by using probabilistic sequence transduction – a probabilistic model widely used in machine translation. TransDroid (Lin et al. 2022) migrates test cases of Web apps to their corresponding Android version, with a Semantic Matching Algorithm similar to CraftDroid. Migrating test cases of the same application across different platforms is generally easier than migrating test cases across different apps.

Many approaches match GUI elements by relying on visual information or DOM structure in the context of test case generation, program repair, and test case execution. Some approaches use visual information to identify the semantic of widgets and screens and to generate test cases. DEEPGUI (YazdaniBanafsheDaragh and Malek 2021) extends Monkey (Google 2017), the standard random approach available in the Android platform. DEEPGUI identifies actionable widgets with deep reinforcement learning, and trains the model with screenshots of GUI states that it obtains by random crawling many applications. Humanoid (Li et al. 2019) trains a deep learning network model with visual information of human-computer interactions, and uses the model to identify the most likely next interactions, and generate meaningful test cases.

Some other approaches repair test cases that broke down in new versions of GUI apps, by modifying the *locators* of the events that cannot retrieve the corresponding widgets. WATER (Choudhary 2011) fixes the broken locators by leveraging DOM structure information. It either relocates the missing widget or finds an element most similar to the missing element. It relocates the missing widget based on attributes of the widget that it retrieves by executing the test case on the old version. It finds an element most similar to the missing one based on either Levenshtein distance of XPaths or other DOM based locators, such as coordinates. WATER works on the syntactic similarity and it is agnostic to the semantic of the attributes. It cannot fix the test case if the widgets are relocated to other pages.

VISTA (Stocco et al. 2018) detects a breakage when the locator of an event either does not return any element or returns a visually different element. It compares visual information of the two versions to find the missing element and repair the test. In this way, VISTA can handle complex breakage scenarios, like new pages added in-between test steps and elements relocated to some neighbor pages. METER (Pan et al. 2022) uses visual information to infer the breakages. It extracts textual information from the GUI with Optical Character Recognition (OCR), and finds a matching widget for the broken locator with both visual and syntactic textual similarity.

GUIDER (Xu et al. 2021) extends METER by using structural data to match the widgets of the broken locators. GUIDER first leverages syntactic similarity of the identifying attributes of widgets and if it cannot find a match, it uses visual similarity.

Yet other approaches use visual scripts to automate the execution of GUI tests by relying on computer vision (Chang et al. 2010; Alégroth et al. 2013). Leotta et al. define PESTO (Leotta et al. 2018) that transforms DOM-based test scripts into visual test script, to reuse GUI tests that are available as DOM-based scripts. RoScript (Qian et al. 2020) transforms videos of users interacting with the application to visual scripts, and uses a physical robot to execute the test script on the device.

Xu et al.’s empirical comparison of GUIDER, METER and WATER (Xu et al. 2021) shows that structural data and visual information are complementary approaches for repairing test case, and combining them together improves the results. The results of Xu et al. support the intuition that combining textual and visual approaches may improve the effectiveness of Test Reuse approaches, and fill the gap between current semantic matching configurations and the perfect Test Reuse. Combining textual and visual approaches is a relevant research direction that can benefit a lot from the results reported in this paper.

## Conclusions

The results presented in this paper indicate that semantic matching is a powerful tool for reusing test cases across applications that share similar functionalities, and shade lights on interesting future research directions. We conclude the paper with some considerations about the practical implications of test reuse, a summary of the impact of the findings reported in this paper on test reuse, and a perspective of the research directions that emerge from the results reported in this paper.

### Practical Implications of Test Reuse

Mobile apps are extremely popular. There are millions of apps in the Google Play app store (AppBrain 2023), and thoroughly testing them is important. Zhou et al. reports that 88% app users would abandon an app if they were repeatedly encountering functionality issues (Zhao et al. 2022). Automatic test generators largely reduce the human effort required for testing, but miss many functionality failures. Reusing test cases across similar apps and functionalities can reveal many functional failures with little human effort if any. Many studies indicate a huge number of similar applications. Ebrahimi et al. classify the 1.8M apps of Apple App Store in 23 categories, and the 2.87M apps of Google Play in 35 distinct categories (Ebrahimi et al. 2021). Thus, effectively reusing test cases across similar applications can largely reduce the human effort required for testing. Developers can complement automatically generated test suites with test cases automatically reused across application, and focus on the few remaining open issues, after an expert assessment of the test results.

### Impact of the Findings Reported in this Paper on Test Reuse

The seminal works on reusing test cases across Android apps exploits semantic matching (Behrang and Orso 2019; Lin et al. 2019; Mariani et al. 2021). This paper presents the first comprehensive study of semantic matching in the context of test generation. Semantic matching is a core component of the approaches that generate test suites by reusing tests across applications that share similar functionalities. Reusing tests can be particularly rewarding in domains with many applications that share similar functionalities, like the fast-growing market of mobile apps. In this paper we study the approaches that reuse tests by migrating test cases across Android apps. We observe that the state-of-the-art approaches share a common workflow that they instantiate with different choices for the main components.

We define a framework to study the impact of the different choices for the components that comprise the Android Test Reuse workflow, and study the choices for the different components both in isolation and in the context of Test Reuse. We present the results of experimenting with 8,099 GUI events from 337 semantic matching configurations We discuss the interesting insights that derive from the experiments, and propose SemFinder, a new Semantic Matching Algorithm to improve Test Reuse. We answer six research questions that investigate the impact of the different components of Test Reuse approaches both in isolation and in the context of test reuse.

The most impactful component on Test Reuse is the Event Descriptor Extractor, since it is the gateway of the necessary information for semantic matching. The impact of the Event Descriptor Extractor grows when used for Test Reuse, with respect to its evaluation in isolation, since the absence of the necessary information results in incorrect matchings, thus the generation of the test case deviates from the relevant windows.

The Semantic Matching Algorithm and Word Embedding have a medium and the Corpus of Documents the lowest impact on semantic matching in the context of Test Reuse. In a nutshell the result suggests a good performance of SemFinder for Semantic Matching Algorithm, WM for Word Embedding, ATM for Event Descriptor Extractor, and Google-Play for Corpus of Documents.

The gap between the best semantic matching configurations and the perfect Test Reuse indicates the important role of the Test Generator. Our manual inspection of the results indicates that the main hurdle that hinders the Test Generator is the incompleteness of the Target Application Model.

When the correct match does not sit in the current window, current approaches hardly locate the correct event because of the lack of information in the Target Application Model. Thus the Test Generator and Target Application Model are the priority for improving Test Reuse.

### Open Research Directions

Our results indicate that there is still a substantial gap between the best semantic matching configurations and the perfect matching. The insights that emerge from our study shade new lights towards new research directions: many-to-many semantic matching, Target Application Model and visual information.

Many-to-many mapping: Ancillary events deal only with simple differences between the cardinality of the source and target tests. In general, the source-to-target test case mappings can be very complex. New many-to-many mappings, like Ermuth and Pradels macro events (Ermuth and Pradel 2016), that is, compositions of multiple events, may largely improve semantic matching.

Target Application Model: Reinforcement learning can be a promising research direction to produce effective Target Application Model to query for the next events.

Visual information:Enhancing textual with visual information can largely improve locating the relevant events, as Xu et al. (2021) empirical study suggests. Leveraging computer vision to take advantage of visual information available in the GUI is the intuitive next research stage.

## Data Availability

The tools developed for this study and the datasets generated and analyzed during the current study are available in the replication repository: https://star.inf.usi.ch/#/software-data/11.

## Appendices

### Mann-Whitney U test

#### MMR

Mann-Whitney U tests of pairs of instances for different distribution (*p-value* $\le 0.05$), according to MMR metrics. The entries with *p-value* $\le 0.05$ are indicated in bold.

### Component C1 Corpus of Documents

**Table:**

	Googleplay	Manuals
Blogs	0.0954	0.3228
Googleplay		**0.0479**

### Component C2 Word Embedding

**Table:**

	es	fast	glove	js	nnlm	use	w2v	wm
bert	0.3188	0.1022	0.0770	0.2670	**0.0125**	**0.0006**	0.0899	**0.0007**
es		0.1519	0.1547	0.2796	**0.0285**	**0.0075**	0.2016	**0.0032**
fast			0.4012	0.3524	**0.0361**	**0.0015**	0.4086	**0.0001**
glove				0.4308	**0.0316**	**0.0027**	0.4291	**0.0001**
js					0.0506	**0.0028**	0.3887	**0.0031**
nnlm						0.1103	**0.0361**	0.1982
use							**0.0011**	0.2718
w2v								**0.0001**

**Table:**

	CraftDroid	intersection	union
ATM_d	**0.0000**	**0.0209**	**0.0000**
CraftDroid_d		**0.0000**	0.3689
intersection			**0.0001**

**Table:**

	ATM	CraftDroid	SemFinder
adaptdroid_a	**0.0000**	**0.0000**	**0.0000**
ATM_a		0.3931	**0.0000**
CraftDroid_a			**0.0000**

### Top1

Mann-Whitney U tests of pairs of instances for different distribution (*p-value* $\le 0.05$), according to Top1 metrics. The entries with *p-value* $\le 0.05$ are indicated in bold.

### Component C1 Corpus of Documents

**Table:**

	Googleplay	Manuals
Blogs	0.1219	0.1293
Googleplay		0.0123

### Component C2 Word Embedding

**Table:**

	es	fast	glove	js	nnlm	use	w2v	wm
bert	0.2427	0.1982	0.1802	0.1778	**0.0046**	**0.0001**	0.2638	**0.0003**
es		0.0735	**0.0478**	0.3886	**0.0028**	**0.0000**	0.0989	**0.0001**
fast			0.4962	**0.0246**	**0.0033**	**0.0000**	0.4030	**0.0000**
glove				**0.0213**	**0.0031**	**0.0000**	0.3675	**0.0000**
js					**0.0004**	**0.0000**	**0.0337**	**0.0000**
nnlm						0.0787	**0.0019**	0.3749
use							**0.0000**	0.0787
w2v								**0.0000**

### Component C3 Event Descriptor Extractor

**Table:**

	CraftDroid	intersection	union
ATM_d	**0.0000**	**0.0004**	**0.0000**
CraftDroid_d		**0.0000**	0.0964
intersection			**0.0005**

### Component C4 Semantic Matching Algorithm

**Table:**

	ATM	CraftDroid	SemFinder
adaptdroid_a	0.4464	**0.0000**	**0.0000**
ATM_a		**0.0048**	**0.0000**
CraftDroid_a			**0.0000**

## Footnotes

1 The framework is available as replication package at https://star.inf.usi.ch/#/software-data/11

2 ATM abstracts attributes into labels, as indicated in the algorithms described above.

3 For sake of notation, we represent the set of events in a test case $\{e_1, \ldots , e_n\}$ with the same symbol used to represent the test case *t*.

4 https://star.inf.usi.ch/#/software-data/11
